# A comprehensive simulation framework for imaging single particles and biomolecules at the European X-ray Free-Electron Laser

**DOI:** 10.1038/srep24791

**Published:** 2016-04-25

**Authors:** Chun Hong Yoon, Mikhail V. Yurkov, Evgeny A. Schneidmiller, Liubov Samoylova, Alexey Buzmakov, Zoltan Jurek, Beata Ziaja, Robin Santra, N. Duane Loh, Thomas Tschentscher, Adrian P. Mancuso

**Affiliations:** 1 European XFEL GmbH, Albert-Einstein-Ring 19, 22761 Hamburg, Germany; 2 Center for Free-Electron Laser Science, DESY, Notkestrasse 85, 22607 Hamburg, Germany; 3 DESY, Notkestrasse 85, 22607 Hamburg, Germany; 4 Shubnikov Institute of Crystallography, Russian Academy of Sciences, Moscow 119333, Russia; 5 The Hamburg Centre for Ultrafast Imaging, Luruper Chaussee 149, 22761 Hamburg, Germany; 6 Institute of Nuclear Physics, Polish Academy of Sciences, Radzikowskiego 152, 31-342 Krakow, Poland; 7 Department of Physics, University of Hamburg, Jungiusstrasse 9, 20355 Hamburg, Germany; 8 Centre for Bio-Imaging Sciences, National University of Singapore, Singapore; 9 Department of Biological Sciences, National University of Singapore, Singapore; 10 Department of Physics, National University of Singapore, Singapore

## Abstract

The advent of newer, brighter, and more coherent X-ray sources, such as X-ray Free-Electron Lasers (XFELs), represents a tremendous growth in the potential to apply coherent X-rays to determine the structure of materials from the micron-scale down to the Angstrom-scale. There is a significant need for a multi-physics simulation framework to perform source-to-detector simulations for a single particle imaging experiment, including (i) the multidimensional simulation of the X-ray source; (ii) simulation of the wave-optics propagation of the coherent XFEL beams; (iii) atomistic modelling of photon-material interactions; (iv) simulation of the time-dependent diffraction process, including incoherent scattering; (v) assembling noisy and incomplete diffraction intensities into a three-dimensional data set using the Expansion-Maximisation-Compression (EMC) algorithm and (vi) phase retrieval to obtain structural information. We demonstrate the framework by simulating a single-particle experiment for a nitrogenase iron protein using parameters of the SPB/SFX instrument of the European XFEL. This exercise demonstrably yields interpretable consequences for structure determination that are crucial yet currently unavailable for experiment design.

The development of X-ray Free-Electron Lasers (XFELs) promises the collection of structural information from very weakly scattering samples that have, until now, eluded investigation by other means. One ambitious goal of XFEL applications is to determine the structure and variability of small biological particles, such as viruses and macromolecules, in their native environment without the need for crystallisation or chemical staining. This paves the way for understanding the structure, dynamics, and function of arbitrary biomolecules, especially those involved in drug design and medical pathology.

Evaluating and optimising the design of XFEL-based single-particle imaging experiments is non trivial, primarily because of the staggering complexity of operating such experiments. In the idealised “diffraction before destruction” imaging scenario^1^, very many copies of a biomolecule are serially illuminated by a stream of intense, femtosecond XFEL pulses. These ultrafast pulses ideally outrun detectable radiation damage in each illuminated biomolecule, and–as they are also ultrabright–can produce enough diffraction signal from each randomly-oriented copy to recover the biomolecule’s three-dimensional (3D) structure *de novo*.

Obtaining accurate biomolecular structures depends heavily on the character and on our control of how the many millions of biomolecules are individually illuminated. These in turn depend on a sequence of design considerations: how consistently the XFEL pulses are produced and shaped; how efficiently these pulses are focused into a sample interaction region; how biomolecules are injected to optimise its illumination rate and quality; the extent and variability of radiation damage in each illumination event, which varies between biomolecules and pulse profiles; how quickly and reliably the faint diffraction signals are recorded; and how to recover statistically-robust structural information from these noisy and incomplete signals. Whereas each of these considerations has been studied in isolation, we still lack a thorough and realistic framework to understand their inter-dependencies, mutual constraints and myriad opportunities for global optimisation across this multi-component experiment. The need for such a framework has been identified by the single particle imaging community as one of the key steps needed to move forward this entire field^2^.

Comprehensive tools to assess the feasibility of XFEL-based imaging experiments are especially important given the stochastic nature of its data acquisition. Evidently, these complex multi-parameter experiments naturally harbour a large phase space of fluctuations. For example, small fluctuations between illumination conditions and the damage profiles of different biomolecules are tolerable if they are uncorrelated enough to be averaged away. For certain conditions and samples, however, this averaging may require an infeasibly large number of measurements. This could be informed by a realistic simulation tool, which may also support the development of adaptive schemes that are better than indiscriminate averaging.

To address these needs of XFEL-based imaging science, we have developed a realistic framework named *simS2E* (simulation from Source To Experiment) that simulates biomolecular imaging at the Single Particles, Clusters and Biomolecules and Serial Femtosecond Crystallography (SPB/SFX) instrument^3^ at the European XFEL^4^,^5^. A flow diagram of the simS2E pipeline is shown in Fig. 1 which consists of six modules: **FEL source** module for simulating source effects in generating femtosecond x-ray laser pulses from electron bunches using FAST^6^ (Section FEL source); **Prop** module for propagation and focusing of the coherent XFEL beam through imperfect optical systems using WPG^7^ (Section Prop); **PMI** module for the interaction of the photons with matter (and the corresponding effect on the scattered photon signal) using XMDYN^8^ (Section PMI); **Diffr** module for the propagation of the scattered signal (including incoherent effects) to the detector plane using SingFEL^9^ (Section Diffr); **Orient** module for determining the unknown orientations of the very many noisy single-particle diffraction patterns using the EMC algorithm^10^ (Section Orient), which are assembled into a three-dimensional (3D) diffraction volume; and **Phase** module for the inversion of this diffraction volume into a 3D electron density of the sample using iterative phase retrieval (Section Phase).

The simS2E framework in Fig. 1 specifies explicit data interfaces so that the actual implementation of each module could be easily replaced. For example, when the modelling complexity of a single module changes, new software or hardware implementations become available, for modelling an XFEL instrument other than SPB/SFX or a source other than the European XFEL, or when comparing the effectiveness of different orientation or reconstruction algorithms.

The following sections will describe each of the six modules aided by a concrete example where we ‘imaged’ nitrogenase iron protein (PDB:2NIP) using two different pulse durations and hence damage profiles: (1) shorter 9 fs full duration at half maximum (FDHM) pulses, and (2) longer 30 fs FDHM pulses. The protein was chosen for its light weight (64 kDa) which pushes the size limit that can be imaged using an XFEL. It also has an iron cluster that has the potential to induce strong photon-matter interactions. Details of the physics in the modelling and simulation parameters are covered in the supplementary sections, including module-specific diagnostics. We discuss the utility of such a framework and conclude with future plans for simS2E, as well as its potential impact on the XFEL-based imaging community.

## FEL Source: X-ray Pulse Generation

As the performance of an FEL is limited by the quality of the electron beam, source simulations are an important aspect of predicting and diagnosing the FEL source. Design parameters, such as the peak brightness and flux, are dependent on the peak current and the required repetition rate. The *FEL source* module simulates the FEL amplification process from the electron gun through the accelerating structures to the undulators, where the radiation is produced. Here we use the FAST code^11^,^12^,^13^ to describe the European XFEL source.

## Prop: Propagation Through the Optics

**Figure 2:**
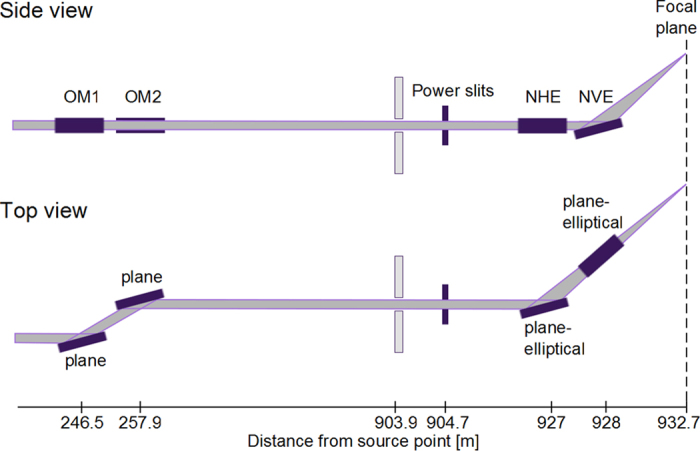
Optical components that were modelled in the SPB/SFX beamline for this work. OMn = offset mirrors, NHE = nanofocus horizontal elliptical. NVE = nanofocus vertical elliptical.

**Figure 3:**
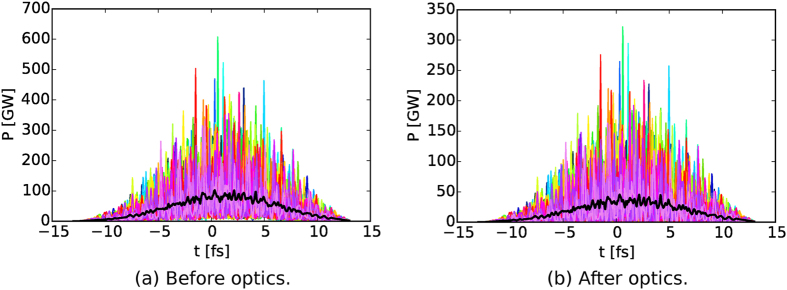
One hundred (100) simulated realisations of the temporal structure of 9 fs X-ray pulses at the SPB/SFX instrument; Instantaneous power (**a**) before and (**b**) after propagation through the optics. Each individual shot is color-coded with an average structure drawn in black. Note that the temporal structure is preserved.

A complete description of the wave field is important for modelling the photon-matter interaction and the quality of scattering patterns. For example, anisotropy in the incident wave field can alter both the photon-matter interaction and the interpretation of the measured diffraction data. The *Prop* module numerically propagates the FEL wave field data at the undulator exit through the beamline optics (see Fig. 2) to the focal plane taking into account the optics’ imperfections (Fig. 3). The WPG package^7^ is used in this module. For further details see the supplementary section.

## PMI: Photon Matter Interaction

The concept of “diffraction-before-destruction” relies on the fact that the X-ray pulses can “outrun” the onset of structural damage–diffraction data measured at the detector represents the structure of the sample before atomic motions due to damage have occurred. A faster and significant contributor to changes in the electron density is electronic damage, and many recent investigations have concentrated on quantifying electronic damage (due to photoionisation, Auger decay and fluorescence) that occurs within the pulse duration^14^,^15^. In fact, for X-rays, the probability that a photon initiates an inelastic event in matter (that causes damage via energy transfer to the sample) is at least one order of magnitude larger than that of the elastic X-ray scattering process carrying the structural information^16^ we seek to exploit. The *PMI* module simulates both the electronic and structural changes of the sample caused by the XFEL pulse using XMDYN^8^.

## Diffr: Diffraction

Diffraction phenomenon lies at the heart of coherent diffractive imaging (CDI) where coherent scattering of photons is interpreted by CDI algorithms. The *Diffr* module simulates the forward scattering of the photons by the sample undergoing radiation damage. SingFEL^9^ is used to simulate coherent (Rayleigh) and incoherent (Compton) scattering by bound- and free-electrons. The addition of incoherent scattering to a single particle imaging simulation is a novel and essential development–the inelastic cross-section dominates for realistic experimental conditions.

**Figure 4:**
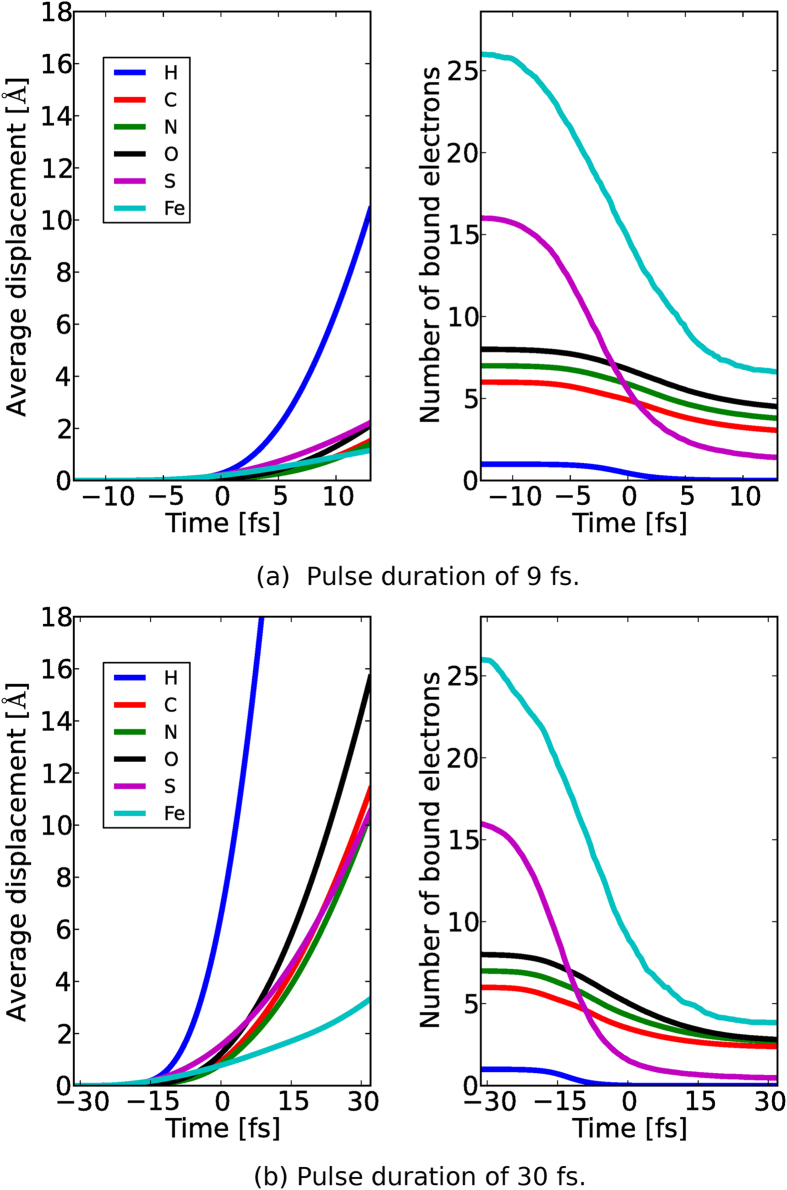
PMI diagnostics for average displacement of atoms (left) and average number of atomic bound electrons (right) for different elements as a function of time during irradiation; (**a**) 9 fs and (**b**) 30 fs cases. The pulse is centred at time 0 fs. Note the difference in average displacement of non-H atoms between the two cases.

**Figure 5:**
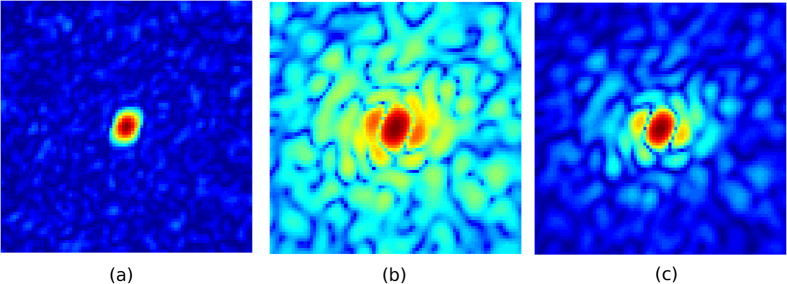
Comparison between the (**a**) instantaneous photon field arriving at the detector at *t* = 64 *fs* (log-scale), (**b**) accumulated photon field without radiation damage (log-scale) and (**c**) accumulated photon field with radiation damage for a 30 fs pulse (log-scale).

In Fig. 4, we see that our sample has already started to Coulomb explode for the 30 fs pulse which results in the loss of speckle contrast in the photon field arriving at the detector at *t* = 64 fs as confirmed in Fig. 5a. The effects of radiation damage is illustrated in the following figure. A comparison between two simulations where (i) no radiation damage effect is included (Fig. 5b), and (ii) which includes radiation damage (Fig. 5c). It is clear that conventional simulations that neglect radiation damage effects yields unrealistically high resolution reconstructions compared to our simulations. It is worth pointing out, however, that the speckle features are mostly conserved in the damage case (even at high-q) suggesting 30 fs pulses may also be short enough for structure determination^17^ given enough photons can be collected at high-q^18^. We attribute this phenomena to the minimal displacement of atoms and low electronic damage for the first half of the pulse followed by a rapid acceleration of the damage process thereafter (Fig. 4b). This demonstrates that the diffraction patterns from single particles (to some extent) also exhibit gating by self-terminating diffraction, similar to nanocrystals^19^.

## Orient: Orientation Recovery

Practical sample delivery technologies^20^,^21^ cause the sample to arrive at the point of interaction at random, unknown orientations. The *Orient* module recovers their orientations using only their diffraction patterns. The challenge here is that these patterns are noisy and only contain a small number of diffracted photons. Here we utilize the EMC algorithm^10^.

**Figure 6:**
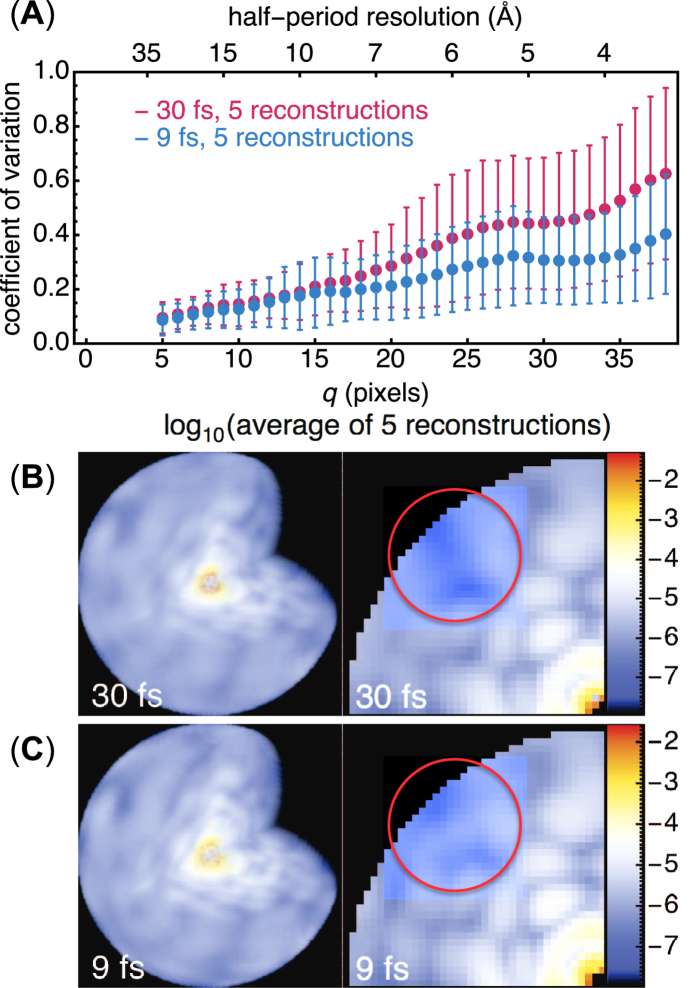
Reconstructed diffraction intensities vary more with longer pulses. We show the coefficient of variation, *σ*(**q** = *q*) in (1), between different reconstructions of the 3D diffraction volume (77^3^ voxels) for the 30 fs and 9 fs cases, both with 200,000 photon patterns containing Compton scattering. (**A**) The twice-averaged *σ*(*q*), once over the five reconstructed volumes in each case then again over resolution shells **q** = *q*, are labelled by filled circles, with error bars showing the standard deviations in this average. There is clearly greater variation in the intensities reconstructed at higher *q* for the longer pulse. (**B**,**C**) Cutaway 3D views (left) and their central sections (right) of the logarithm of their average reconstructed intensities. Loss in speckle contrast at higher spatial frequencies (encircled in red) is more severe with longer pulses.

To test if our recovered 3D diffraction volumes are self-consistent and repeatable, we compared five reconstructions from random starting points. These 3D volumes were mutually aligned, from which we computed the following spatial frequency **q**-resolved coefficient of variation between these *n* aligned 3D volumes *I*_*n*=1…5_(**q**),


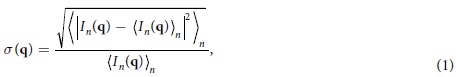


where 〈…〉_*n*_ denotes averages over the *n* volumes. These variations are then averaged azimuthally along resolution shells to produce the figure-of-merit in Fig. 6, juxtaposed with a planar section of their averaged 3D diffraction intensities. Simply put, this coefficient of variation *σ*(**q**) quantifies how much unbiased reconstructions of the 3D diffraction volume depart from each other as a function of resolution.

A longer pulse duration expectedly causes larger differences in sample damage trajectories, and hence larger intensity variations - even if their diffraction patterns were perfectly oriented in the 3D volume. Because orientations are recovered from these intensities, these variations also cause larger misorientations. These two factors combined ultimately reduce the average contrast and our certainty of higher resolution speckles in the reconstructed diffraction volume (Fig. 6).

## Phase: Phase Retrieval

To recover the scatterer’s three-dimensional (3D) electron densities, we must retrieve the phases complementary to the Fourier amplitudes measured on the photon detector. In biological single-particle imaging, these phases are computationally retrieved with algorithms that apply two known properties of the scatterer^22^,^23^,^24^: (1) its 3D Fourier transform must agree with the assembled 3D diffraction intensities in Fig. 6, and (2) the scatterer’s electron density is positive and lies wholly within a finite spatial extent *S*. The final *Phase* module reconstructs the scatterer’s electron density with the Difference Map algorithm^23^, modified to be more robust with noisy and incomplete data^25^,^26^.

**Figure 7:**
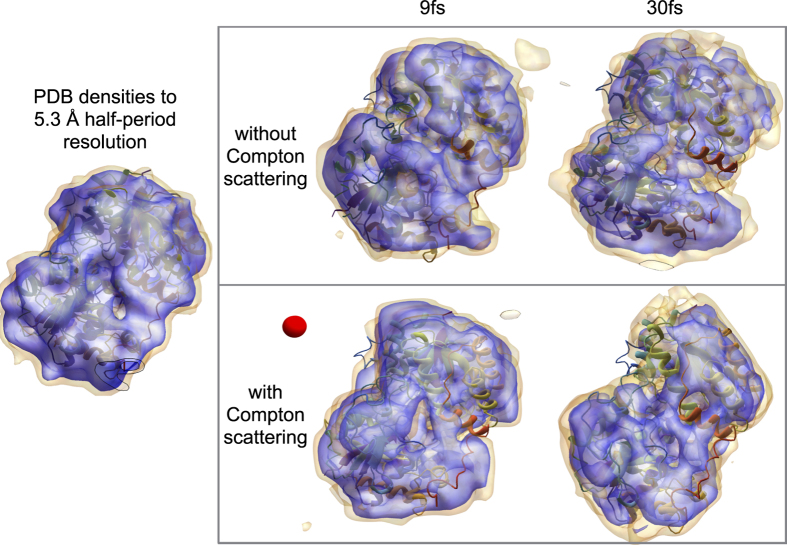
The reconstructed electron density of the nitrogenase iron protein with the known structure (ribbon rendering), and a 7 Å-diameter reference red sphere. Electron densities of the average reconstruction at the 5% and 15% levels (yellow and purple, respectively) are rendered in all cases. The protein’s low-resolution features were recovered in all four cases, with surface electron densities showing the most variation and hence least certainty. Loss of reproducible density is more severe in the 30 fs case due to greater damage. Degradation of surface contrast is expected in previous damage-only simulations and may, in future studies, be tampered by a sacrificial water layer^44^. Compton scattering reduces speckle contrast (Fig. 6), limiting our ability to correctly determine the support needed for robust iterative phase retrieval.

The Phase-Retrieval Transfer Function (PRTF) is typically used to estimate the reproducibility of the electron densities in iterative phase retrieval^27^. Each reconstruction’s PRTF, however, does not indicate its resemblance to the undamaged structure. Here we tested four cases with *simS2E* as shown in Fig. 7: 9 fs and 30 fs, with and without Compton scattering. Although the full-period resolution in the electron density of these four cases were all 7.2 Å at half-PRTF (each averaged over five hundred randomly initialized reconstructions), reconstructions with 9 fs and 30 fs pulses in Fig. 7 had different damage profiles. Specifically, while the loss of reconstructed electron density on the particle’s surface occurs even with only elastic scattering, it is exacerbated by Compton scattering and longer pulses.

## Methods

### FEL source amplification

In our case study, we chose to simulate two pulse durations at 5 keV photon energy; 9 fs and 30 fs at full duration at half maximum (FDHM), with an accelerator electron energy of 12 GeV using FAST^11^. Note that the full durations for the 9 fs and 30 fs pulses are around 22.93 fs and 76.43 fs. Two sets of statistically independent FEL pulses in the non-linear, over-saturated mode were generated, for electron bunch charges of 100 and 250 pC, respectively. FAST takes into account all important physical effects such as diffraction of radiation, slippage of radiation (time-dependent effects), space charge, emittance (betatron oscillations), and energy spread in the electron beam. We generated 55 instances of the pulse for each case. The average number of photons per pulse was 13 ± 0.25 × 10^11^ for the 9 fs pulse and 30 ± 0.5 × 10^11^ for 30 fs pulse. The values in the Supplementary Table S1 are for saturation (active undulator length z = 60 m). We used over-saturated data (z = 106 m) with more photons per pulse, see also Supplementary Fig. S2.

### Propagation through the optics

In our case study, the beamline optics at the SPB/SFX instrument (Fig. 2) consists of a pair of 0.8 m long offset mirrors located 245 m from the SASE1 undulator source, with an incidence angle of 3.5 mrad, and a focusing Kirkpatrick-Baez (KB) system of two 0.95 m long plane elliptical focusing mirrors located 955 m from the source, the horizontal mirror with focal distance 3 m, and the vertical mirror with focal distance 1.9 m, both with 3.5 mrad incident angle. To quantify the influence of mirror imperfection, on these under construction optics, our model used the expected surface profiles with better than 2 nm peak-to-valley residual height errors based on real metrology data from similar mirrors^28^.

According to the simulation, the beam at the sample is focused to 250 × 160 *nm*^2^ at FWHM delivering 5 × 10^11^ photons per pulse. The shot-to-shot instantaneous power of the 9 fs X-ray pulses is shown in Fig. 3. The temporal fluctuations in beam intensity have a major influence on the damage process and we observe that the propagated pulse inherits the source pulse temporal structure, however, the pulse energy decreases by a factor of 2.3, mainly due to aperturing of the relatively large 5 keV beam by the mirrors’ size. The focus size is 3 times larger than a 100 *nm*^2^ focus which is often assumed in simulations.

### Photon matter interaction

As the next step, we irradiate a nitrogenase iron protein (PDB:2NIP) with the simulated, time-dependent X-ray pulses from the *Prop* module for both the 9 fs and 30 fs cases. We utilized XMDYN^8^, a simulation toolkit developed for modelling the dynamics of matter induced by intense X-ray irradiation which has already been successfully used to interpret high intensity spectroscopy measurements^8^,^29^,^30^ as well as low intensity (synchrotron) data^31^. The code provides a fully microscopic description of the dynamics of the sample and therefore gives access to all of its imaging related properties, such as to the electronic states and real space positions of the atoms. The electronic structure is described in the atomistic approximation: the occupancy of the atomic orbitals of all the atoms are followed individually using Monte Carlo technique, realizing an ionisation path of the system. Relevant atomic parameters are calculated by the XATOM package^14^. The real space dynamics of atoms, ions and electrons released from the atoms in ionisation events is tracked using classical Molecular Dynamics.

We generate stochastic realizations of the temporal sample evolution (sample damage) and output the real space positions of the atoms, the form factors and the structure factors for incoherent scattering from the bound and free electrons^14^, separately (Further details are given in Supplementary Section ‘Photon matter interaction’).

According to our simulations, approximately 3500 photoionisation events happen on average in the investigated sample during a single shot. Applying the usual radiation damage quantification, when all the energy of the absorbed photons is assumed to be kept and distributed within the sample, it corresponds to 25 GGy of radiation dose to the 4,735 non-hydrogen atom large molecule. However, we should note that this dose definition is not accurate for finite molecular samples as significant part of the photon energy absorbed is taken away by energetic photoelectrons escaping from the sample.

The sample undergoes significant electronic damage where the light atoms lose around half of their electrons and the heavier ones such as sulphur and iron are stripped of almost all electrons within the pulse duration as shown in Fig. 4a,b. The average displacements of non-hydrogen atoms for a 9 fs pulse are restricted to 2 Å deviations within the pulse duration (Fig. 4a). On the other hand, the 30 fs pulse on average displaces the ions many times the imaging resolution (Fig. 4b) which suggests that the Coulomb explosion^18^ has already occurred. The radiation damage is so significant, even for short pulses, that any simulation neglecting such effects would produce unrealistically optimistic results (see Supplementary Section ‘Phase retrieval’ for an explicit comparison).

### Diffraction

**Figure 8:**
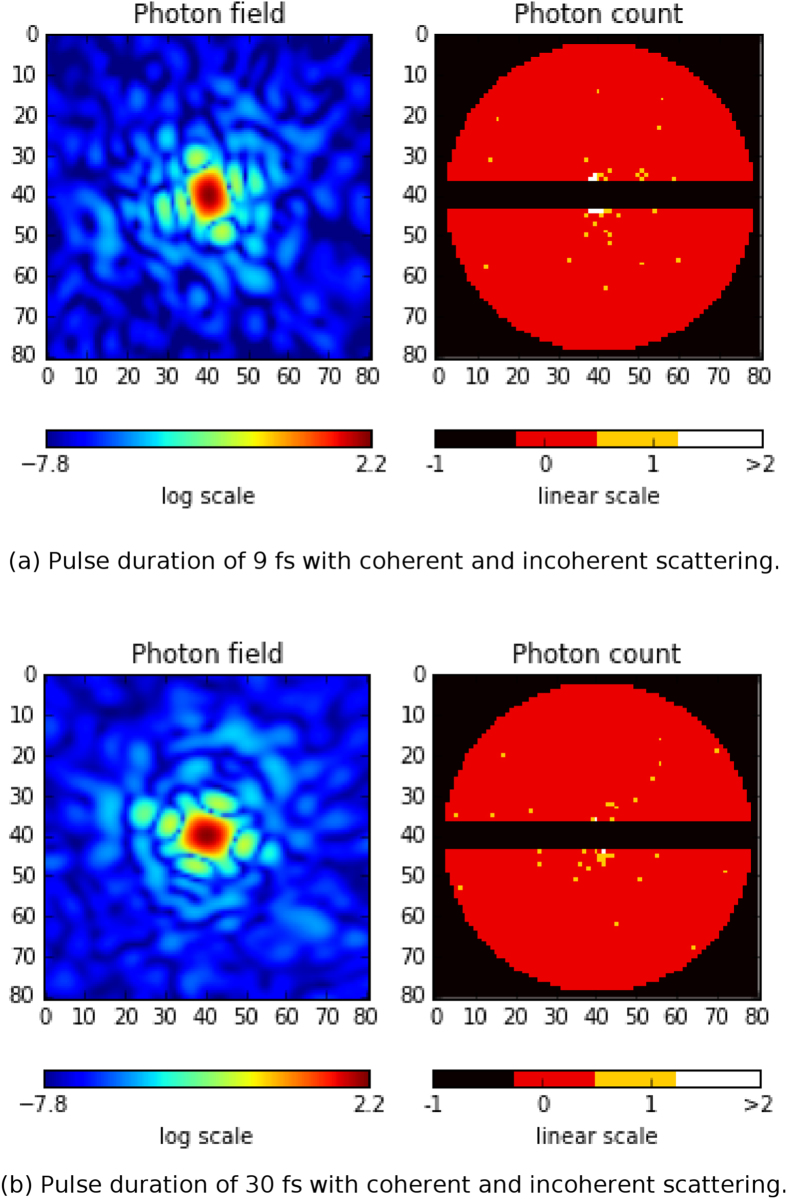
Simulated diffraction patterns for the (**a**) 9 fs pulse, (**b**) 30 fs pulse with coherent and incoherent scattering.

**Figure 9:**
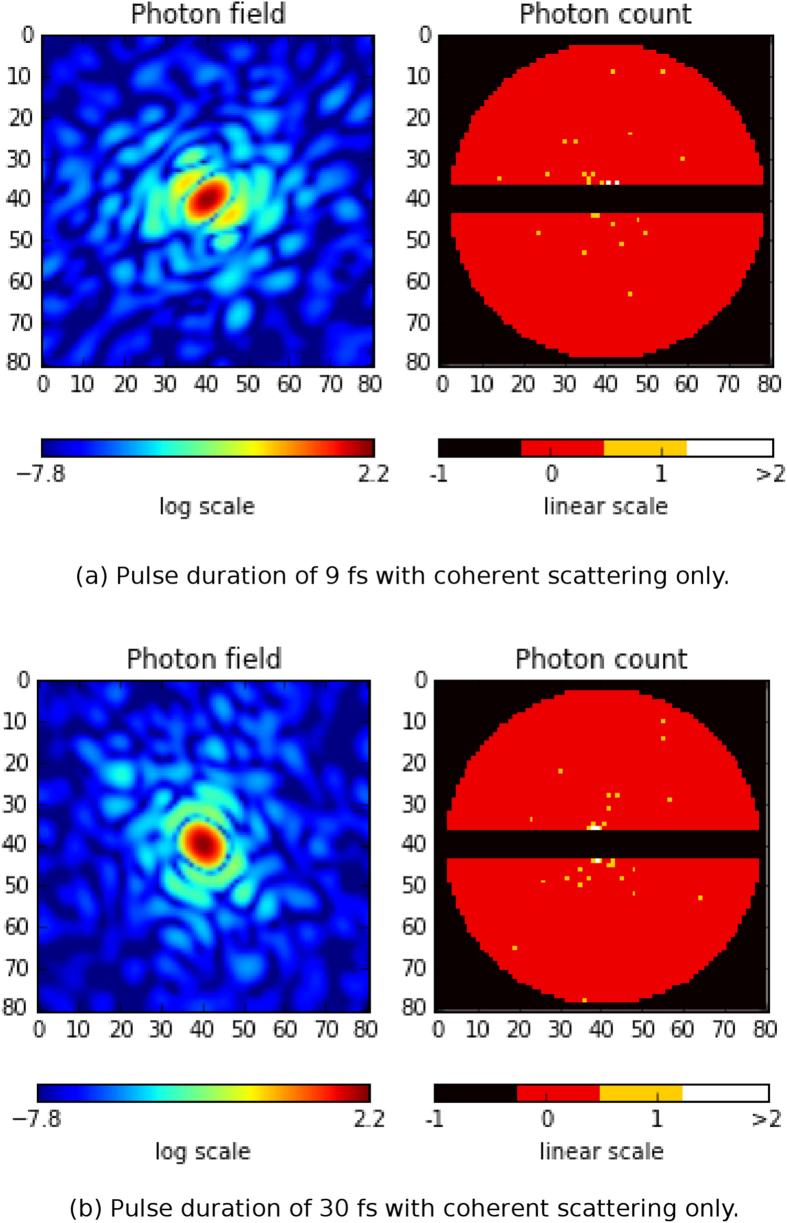
Simulated diffraction patterns for the (**a**) 9 fs pulse, and (**b**) 30 fs pulse with coherent scattering only.

We simulated four datasets each consisting of 200,000 diffraction patterns; 1) 9 fs FDHM pulses with coherent and incoherent scattering, 2) 30 fs FDHM pulses with coherent and incoherent scattering, 3) 9 fs FDHM pulses with coherent scattering only, and 4) 30 fs FDHM pulses with coherent scattering only. The time-dependent form factors from the *PMI* module and the beam intensities from the *Prop* module are used to calculate the diffraction patterns using SingFEL^9^. Each diffraction pattern is a summation of all instantaneous (coherent and incoherent) scattering fields produced by the sample undergoing radiation damage weighted by the instantaneous beam intensities. Further details are given in the supplementary section 1.4. Examples of the resulting diffraction patterns for the two pulse durations with and without incoherent scattering contribution are shown in Figs 8 and 9, respectively. The measured diffracted intensities with a 7 pixel detector gap results in an average photon count of around 190 photons/frame. Although incoherent scattering is relatively weak, it is radially isotropic which means that it will persist when compressed into a 3D diffraction volume and potentially introduce problems for phase retrieval (which considers only the coherently scattered radiation as signal).

### Orientation recovery

Several algorithms can assemble weak individual diffraction patterns (Fig. 8) into a three-dimensional (3D) diffraction volume by recovering the relative orientations between pairs of patterns. They mostly use either semi-empirical distance functions^32^,^33^,^34^,^35^,^36^,^37^ or known noise distributions^10^,^38^ to determine if one pattern is orientationally close to or far away from another. By default, the *Orient* module uses the EMC algorithm^10^,^39^,^40^ detailed in the supplementary section 1.5, which reconstructs a 3D diffraction volume *de novo* by iteratively maximising the likelihood that it produces a given set of photon-limited diffraction patterns. Because this maximisation reconstructs a distribution of orientations for each pattern, we can also obtain each pattern’s most likely orientation. We applied EMC using the Orient module to our example cases.

### Phase retrieval

The key to successful phase retrieval is an accurate support *S* that describes the spatial extent of the sample and reduces the search space for the correct set of phases. This is obtained by iteratively shrinking an initial support *S*^(0)^ determined from the autocorrelation function, which is in turn derived from the Fourier transform of the 3D intensities in Fig. 6 that is, information that can be obtained from measured diffraction data alone. This support is iteratively modified *S*^(*n*≥1)^ using a variant of the shrinkwrap algorithm^41^ while maintaining a healthy search for phases. To update the support from *S*^(*n*)^ → *S*^(*n*+1)^, five hundred randomly initialized reconstructions most compatible with the current support *S*^(*n*)^ and Fourier intensities (selection criteria from^42^) are averaged, thresholded, then blurred by a 5 × 5 × 5-voxel kernel.

## Discussion

In our example, we modelled SASE pulses in time and space using FAST code and then propagated them through the focusing optics using WPG. Protein samples (PDB:2NIP) were then irradiated with the focused beam using XMDYN and the diffraction patterns were generated using SingFEL for four different scenarios. The random orientations of the 200,000 diffraction patterns were composed into a single diffraction volume using the EMC algorithm^10^. This corresponds to four continuous 12-hour shifts at LCLS with a 1% particle hit rate, and is equivalent to 2 hours at the European XFEL at 3500 Hz detector-limited rate.

The *simS2E* framework has allowed us, in a detailed simulation, to explore the differences between illuminating a biomolecule with realistic ensembles of either 9 fs pulses and 30 fs pulses while keeping all other experimental conditions constant. In particular, the addition of incoherent scattering to the model (and comparing the case with it artificially removed) adds an essential authenticity to the entire simulation pipeline. That the longer pulses cause more radiation damage to the sample and impact the fidelity of reconstruction more than the shorter pulses is unsurprising, however, Fig. 7 also suggests that reconstructions (even for longer pulse durations) may improve if photon detectors had energy selection (via hardware or software) that can promote the ratio of elastic to inelastic scattering. Shorter pulses may also improve reconstruction quality, but will require more patterns to accumulate enough total photons.

The utility of the *simS2E* framework extends beyond our simplistic four-case demonstration. We can now systematically explore the effects of changing meaningful parameters in a simulation to determine if an imaging experiment is feasible. This tool opens the door to a plethora of comparative studies including exploring different orientation and phase-retrieval algorithms, comparing alternative focusing schemes, or mapping out different source parameters (such as photon energy, source size, pulse energy or pulse duration) and in the future comparing the effect of different detector noise and background levels.

More improvements are being made to the *simS2E* framework in the near future. Work on a detailed, validated detector simulation toolkit, X-CSIT, is currently in progress. Preliminary validation has been performed for pnCCD- and LPD-type detectors^43^ and initial integration tests with simS2E framework have been successful. Additionally, an injector module may be useful for modelling the particle distribution in space to develop hit-finding algorithms. A veto module could be added to measure other signatures of a “hit”; such as ion time-of-flight and fluorescence, providing an even higher degree of sophistication to the framework. These additional data interfaces and module executables will be made available to the public.

## Conclusion

We have presented the *simS2E* framework for simulating a single particle imaging experiment at an FEL facility, including source parameters, propagation of the coherent X-rays through optical elements, interaction of the photons with matter, and their subsequent detection and structure determination. To demonstrate this framework, we showed a single-particle structure determination example using parameters of the SPB/SFX instrument at the under-construction European XFEL.

The realistic test cases presented here validated our expectations that structure determination is adversely impacted with longer x-ray laser pulses and incoherent scattering. More importantly, our simulations identified where the impact is expected to occur in different scenarios. Our framework accommodates complex experimental scenarios to help identify critical regions of parameter space needed for single particle imaging to succeed, and hence direct efforts to best utilize these next generation light sources and their very valuable beam time. The present framework’s documentation, executables and data are publicly available and maintained on www.xfel.eu/simS2E.

## Additional Information

**How to cite this article**: Yoon, C. H. *et al.* A comprehensive simulation framework for imaging single particles and biomolecules at the European X-ray Free-Electron Laser. *Sci. Rep.* **6**, 24791; doi: 10.1038/srep24791 (2016).

## Supplementary Material

Supplementary Information

## Figures and Tables

**Figure 1:**
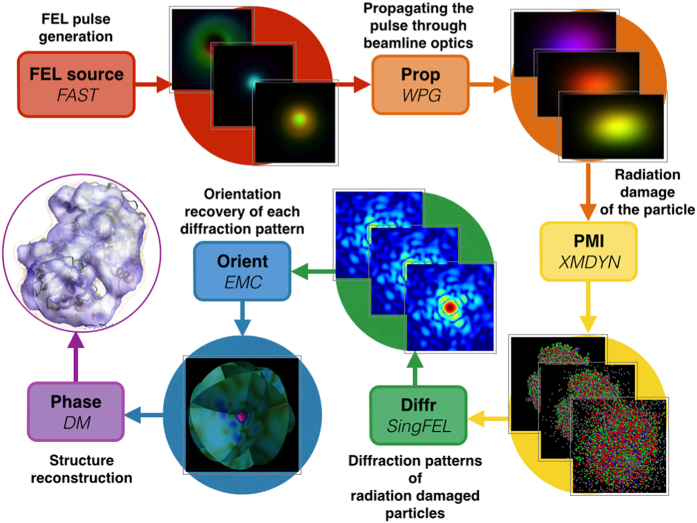
Flow diagram of the simS2E pipeline from XFEL pulse generation to the electron density reconstruction. The **FEL source** module simulates the electron bunch exiting the undulators using a software package called *FAST* (Section FEL source). The simulated electron bunch is propagated through the beamline optics to the interaction point using the **Prop** module using *WPG* (Section Prop). The **PMI** module simulates the radiation damage of the protein sample when it is irradiated by the simulated laser pulse using *XMDYN* (Section PMI). The **Diffr** module simulates the time-averaged diffraction pattern of the radiation-damaged protein using *SingFEL* (Section Diffr). The **Orient** module recovers the orientation of the individual diffraction patterns using *EMC* (Section Orient) and the **Phase** module solves the phase problem to recover the electron density using *DM* (Section Phase).
