# Supplementary material for: A comprehensive simulation framework for imaging single particles and biomolecules at the European X-ray Free-Electron Laser
Source: Sci Rep. 2016 Apr 25;6:24791. doi: 10.1038/srep24791 (PMC4842992; doi:10.1038/srep24791)
Supplement: Supplementary Information [file srep24791-s1.pdf]

Supplementary material: A comprehensive simulation framework for imaging single particles and biomolecules at the European X-ray Free-Electron Laser

Chun Hong Yoon*, Mikhail V. Yurkov, Evgeny A. Schneidmiller, Liubov Samoylova, Alexey Buzmakov, Zoltan Jurek, Beata Ziaja, Robin Santra, N. Duane Loh, Thomas Tschentscher, Adrian P. Mancuso*

FEL source amplification

The FEL source module is implemented using FAST which is a set of codes for analysing the FEL amplification process in the framework of 1D and 3D models using different techniques described in.[1–5] Analytical techniques implemented in these codes allow one to analyse beam radiation modes (eigenvalue equation), and the amplification process in the linear stage of amplification (initial-value problem). Numerical simulation codes allow one to simulate the FEL process using both steady-state and time-dependent models. An approximation of a uniformly focussed electron beam is used. The field solver uses an expansion of the radiation in the azimuthal modes. Calculation of the radiation fields is performed using retarded potentials. The code can simulate amplification processes in helical and planar undulators. Simulation of higher odd harmonics in the planar undulator geometry is also possible.[5] The code has been thoroughly tested in the high gain exponential regime using analytical results for the beam radiation modes (complex eigenvalues and eigenfunctions).[1–3] Start-up from shot noise and the linear stage of amplification in FAST has been also tested using three-dimensional analytical results for the initial-value problem[4] which can also be simulated with an artificial ensemble,[6,7] and with tracing the actual number of electrons in the beam.[8] The latter option is straightforward and transparent: the simulation procedure corresponds to real electrons randomly distributed in full 6D phase space. This allows us to avoid any artificial effects arising from standard procedures of macroparticle loading as described in.[8]

The baseline parameters of the European XFEL have undergone several revisions: in 2006, 2010, and 2014.[9–13] The operating range for bunch charge is from 20 pC to 1 nC, peak current is 5 kA, and normalized rms emittance is between 0.3 mm-mrad and 1 mm-mrad depending on the bunch charge. The tunability range of SASE1/2 undulators, in terms of the undulator parameter, is 1.65 - 4 ($\lambda_{\mathrm{max}}/\lambda_{\mathrm{min}} = 3.5$). Supplementary Fig. S1 shows the operating range of SASE1/2 in terms of the radiation energy and the number of photons in the pulse for a bunch charge of 0.1 nC. The operating wavelength range is defined by the operating range of the undulator gap and the requirement of saturation at the full undulator length. Note that both quantities are scaled approximately proportionally to the bunch charge. Four electron energies are fixed at preferred operating points: 17.5 GeV, 14 GeV, 12 GeV, and 8.5 GeV that provide the most flexibility for conducting experiments.[14]

The choice of the operating point for imaging experiments is the subject of compromise between the number of photons, radiation wavelength, pulse duration, and coherence properties of the radiation.[15–17] The number of photons per pulse varies by an order of magnitude, from about 10^{11} at 0.1 nm radiation wavelength and up to 1.4×10^{12} at 0.6 nm radiation wavelength for a 100 pC electron bunch charge. The pulse duration and the number of photons scale approximately proportionally to the bunch charge. The dependence of the number of photons on the electron energy is rather weak. The spatial coherence of the radiation improves for higher electron energies and longer wavelengths. On the other hand, resolution of image reconstruction decreases with the increase in radiation wavelength.

For this case study, we fixed the photon energy to 5 keV and operate at the 12 GeV point in the electron energy. Simulations have been performed for two sets of the electron beam baseline parameters corresponding to a bunch charge of 100 pC and 250 pC.[18] FAST produces a three-dimensional array of the radiation field which can then be traced by the propagation module through the beam optics. Photon beam properties in saturation for the bunch charge of 100 pC are compiled in Supplementary Table S1. The photon energy specified corresponds to the 'resonant wavelength' used as the FAST input parameter.

The evolution of pulse energy along the undulator is shown in Supplementary Fig. S2 at three operating points for various bunch charges. The best coherence can be achieved at the saturation point (about 60 m), but the simulation was conducted at the over-saturated point (106 m) for higher fluence.

The temporal and spectral structure of the radiation pulse for a single shot is shown in Supplementary Figs. S3 and S4, respectively. When we trace the amplification process beyond saturation, we observe a lengthening of the radiation pulse. This happens due to two effects: First, parts of the electron bunch with small current start to bring the FEL process into the saturation regime in the longer undulator. Second, slippage of the radiation takes place leading to lengthening of the radiation pulse. While the energy in the radiation pulse continues to grow, the coherence time drops as we can see from the spectrum broadening in Supplementary Fig. S4. The degree of transverse coherence drops as well.[8]

Table 1. Photon beam properties in the saturation for SASE1 at the European XFEL

Parameter	Units	Value
Energy of electrons	GeV	12
Bunch charge	nC	0.1
Radiation wavelength	nm	0.25
Photon energy	keV	4.96
Pulse energy	mJ	0.4
Peak power	GW	45
Average power	W	11
FWHM spot size	μm	37
FWHM angular divergence	μrad	3.7
Coherence time	fs	0.25
FWHM spectrum width, dw/w	%	0.24
FWHM radiation pulse duration	fs	9
Number of longitudinal modes	#	37
Fluctuations of the pulse energy	%	5.5
Degree of transverse coherence	#	0.9
Degeneracy parameter	#	1.3×10^{10}
Number of photons per pulse	#	5×10^{11}
Average flux of photons	phot./sec	1.4×10^{16}
Peak brilliance*	#	1.4×10^{33}
Average brilliance*	#	3.5×10^{23}

*Units of phot./sec/mm^2/rad^2/0.1% bandwidth.
Averaged characteristics are calculated for 27,000 pulses per second.

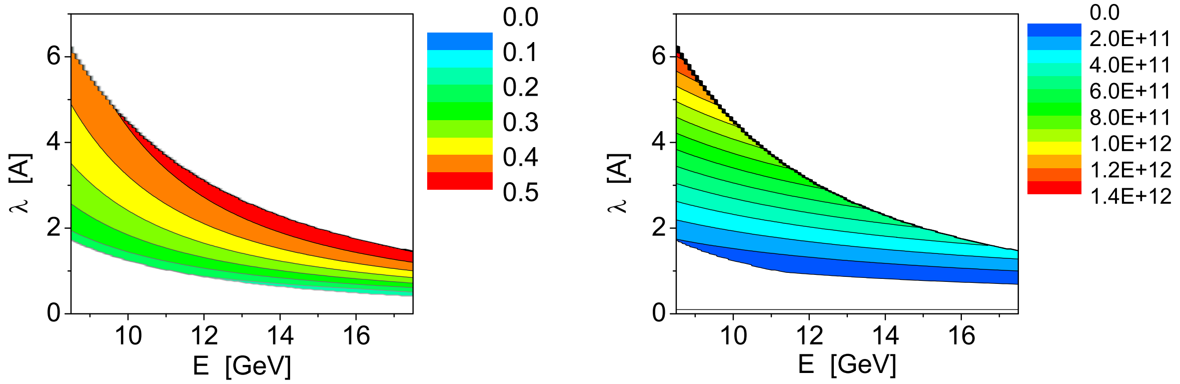

(a) Pulse energy.

(b) Number of photons.

Figure 1. An overview of the photon beam properties of the European XFEL for SASE1/2 in the saturation with bunch charge at 0.1 nC; (a) contour plot of the pulse energy, and (b) contour plot of the number of photons in the pulse. Units for the pulse energy and brilliance are in mJ.

Propagation through optics

SASE FEL radiation has an almost full transverse coherence, such that wave optics is a natural and reliable way to deal with interference effects. The wavefront simulations presented here were performed using the near field approach implemented in the Synchrotron Radiation Workshop (SRW) software package.[19] For high-level Python based access to SRW functions, the WPG framework[20] was used that also includes the HDF5 interface support and set of XFEL-specific routines.

For small emission and observation angles, the free space propagation of the transverse components in the frequency domain from a point $\mathbf{r_1}$ towards a point $\mathbf{r_2}$ can be described in terms of the Huygens-Fresnel principle by an integration over the

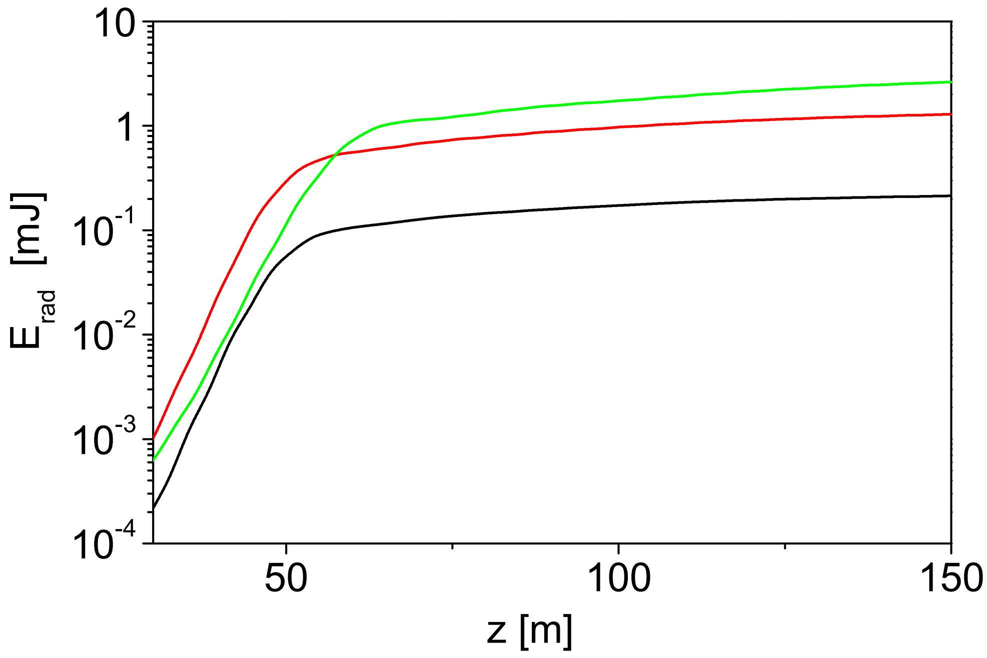

Figure 2. Average energy in the radiation pulse versus undulator length. Color codes (black, red, and green) refer to the bunch charges at 20 pC, 100 pC, and 250 pC, respectively. Electron energy is 12 GeV. Radiation wavelength is 0.25 nm.

plane Σ_1 perpendicular to the z-axis

$$\mathbf{E}_\perp(\mathbf{r_2},\omega) \approx \frac{-ik}{2\pi z}\int\int e^{\frac{ik}{2z}[(x_2-x_1)^2+(y_2-y_1)^2]}\mathbf{E}_\perp(\mathbf{r_1},\omega)d\Sigma_1. \quad (1)$$

where Σ_1 is a plane of the initial wavefront, $d\Sigma_1 = dx_1dy_1$, $|r_2 - r_1| = \sqrt{\Delta z^2 + (x_2-x_1)^2 + (y_2-y_1)^2]}$, and $k = \omega/c$ is the wave vector. This can be numerically solved by means of a 2D FFT. This approach was successfully applied for modelling and prediction of FEL wavefronts after propagating through real grazing incidence mirrors and CRLs. A good agreement with experiment was observed, for instance, in ref.[21]

Beamline components, such as apertures, absorbers, CRLs, and ultra-smooth mirrors with residual height errors, can be described as complex, thin optical-element propagators:

$$\mathbf{E'}_\perp(x,y,\omega) = P(x,y,\omega)\mathbf{E}_\perp(x,y,\omega) \quad (2a)$$

$$P(x,y) = A(x,y,\omega)\exp[if(x,y,\omega)], \quad (2b)$$

where the amplitude $A(x,y)$ corresponds to an aperture/obstacle mask or absorption in an absorber plate and $f(x,y)$ corresponds to the modification of the wave field phase. For simulation of focusing elliptical grazing incidence mirrors, a thick element propagator based on the local stationary-phase approximation[22] was recently included in SRW package and used in the *Prop* module. Wavefront distortions caused by the grazing incidence mirror's residual height errors, known from surface metrology measurements, can be taken into account in form of an achromatic phase screen:

$$f(x,y,\omega) = -\frac{2\pi}{\lambda}2\Delta h(x,y)\sin\theta, \quad (3)$$

where θ is the grazing angle at the mirror, and $\Delta h(x,y)$ are the residual surface height errors provided by metrology measurements.

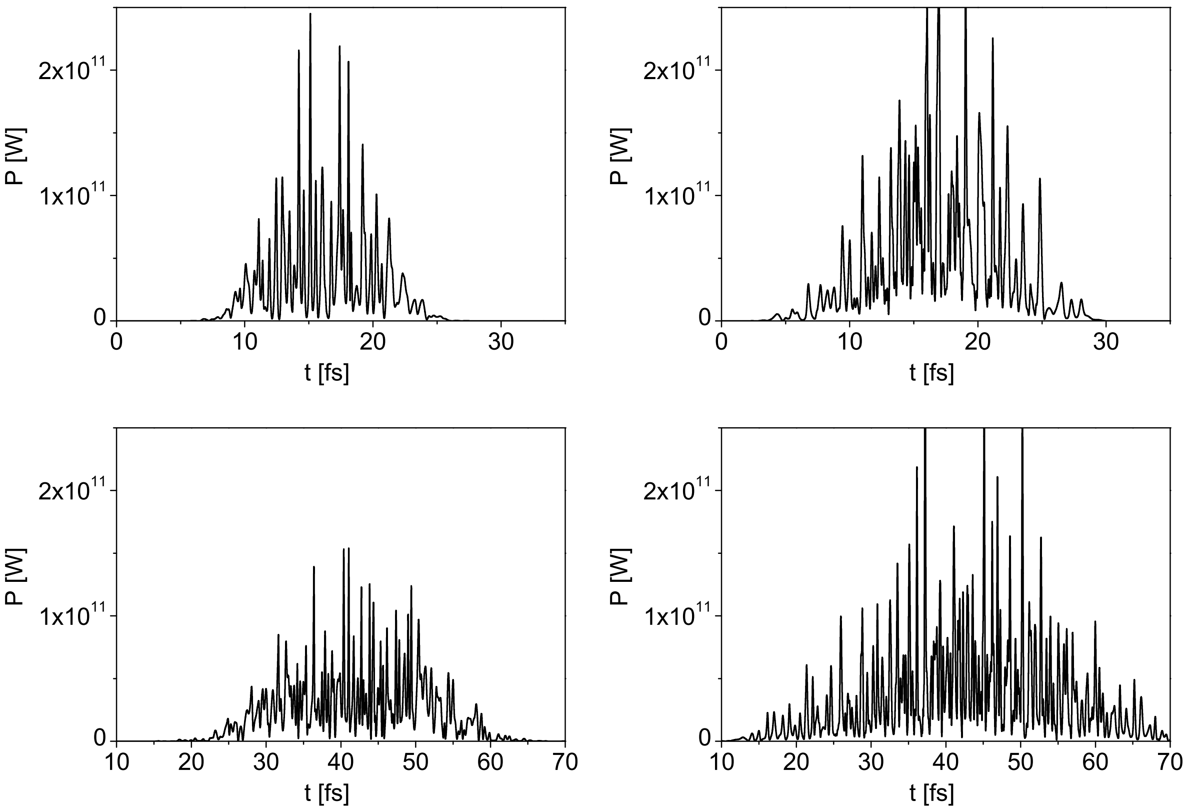

Figure 3. Temporal structure of a single pulse at different stages of amplification. The pulse at the saturation point (Undulator length = 60 m) is shown on the left column. The pulse at the over-saturated point (106 m) is shown on the right column. Top and bottom rows represent bunch charges at 100 pC (9 fs pulse) and 250 pC (30 fs pulse), respectively. Electron energy is 12 GeV. Radiation wavelength is 0.25 nm.

For the over-saturated mode that we used in simulations, the coherence time was about 0.2 fs for 9 fs pulse duration and about 0.25 fs for 30 fs pulse. As one can see in Supplementary Fig. S5, the beam parameters after propagation through the beamline preserve the main features of the mother pulse, while the beam centre of mass is focused down to a sub-micrometer spot, matching well the size of a typical object. The X-ray intensity in the focal spot at the sample is smooth, symmetric and very uniform along the pulse (Supplementary Fig. S6). In contrast, the phase of the wavefront varies in a random manner along the pulse duration and may also occasionally display a weak variation in the transverse direction.

Photon matter interaction

For implementing the photon matter interaction module, we utilized XMDYN,[23] a simulation tool developed for modeling the dynamics of matter induced by intense X-ray irradiation which has already been successfully used to interpret high intensity spectroscopy measurements[23–25] as well as low intensity (synchrotron) data.[26] XMDYN is a Monte Carlo–Molecular Dynamics based particle approach. One run generates temporal snapshots of a single trajectory which is a realization of the temporal evolution of the system affected by stochastic damage processes. The initial sample is described as a set of individual atoms in their neutral ground state as described by the Protein Data Bank (PDB) file. The hydrogen atoms, missing in the PDB file, are added using the 'openbabel' package.[27,28] Afterwards, a random orientation in SO(3) is enforced on the whole molecule. The dynamics of the bound electrons are not calculated. Instead, the occupation number of the atomic orbitals is tracked (following all intermediate states) for each atom. The electronic configuration can change due to stochastic ionization or inner shell relaxation events. XMDYN takes into account photoionization and all possible Auger and fluorescent decay channels for a given electronic configuration by assigning probability rates to the processes. The corresponding decays are exponential in time, but only one of them occurs in a specific realization. It is chosen based on random number generation (Monte Carlo block). The required physical parameters (cross section and rate data) are calculated with XATOM,[29] an integrated toolkit for X-ray and atomic physics applying the ab-initio Hartree-Fock-Slater model.[29,30]

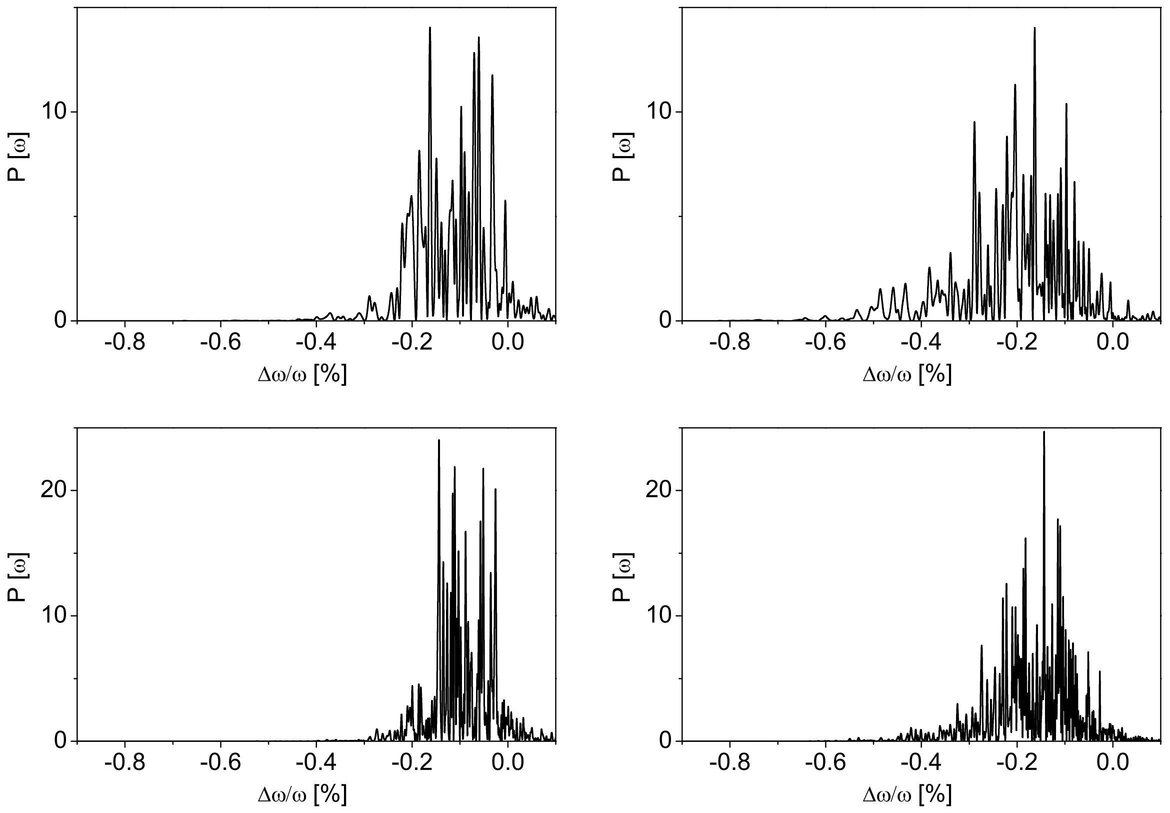

Figure 4. Spectral structure of a single pulse at different stages of amplification. The pulse at the saturation point (Undulator length = 60 m) is shown on the left column. The pulse at the over-saturated point (106 m) is shown on the right column. Top and bottom rows represent bunch charges at 100 pC (9 fs pulse) and 250 pC (30 fs pulse), respectively. Electron energy is 12 GeV. Radiation wavelength is 0.25 nm.

The real space dynamics of the atoms, ions and electrons ejected from the atoms are modelled using classical Newton's equations (Molecular Dynamics block). Coulomb forces act between charged particles. Chemical bonds are neglected between non-hydrogen atoms. This approximation is accurate enough if the sample is intensely ionized within a short time interval.[23] The equations of motion are solved with a multiple-time-step Position Verlet algorithm[31] using sub-attosecond timesteps. Free electrons can further ionize atoms and ions via secondary (impact) ionization. The cross sections are calculated with the Binary-Encounter-Bethe model.[32] While the main channels of ionization for the non-hydrogen atoms are photoionization and Auger decay, for hydrogen these processes are negligible. On the other hand, charge migration effects may remove the (only) electron from hydrogen if it is close to a highly charged ion. Therefore we assume a priori that the electron of a hydrogen was transferred promptly to a neighboring ion having equal to or higher than +2 charge.

Snapshots of the time evolution are recorded. They contain all atomic positions and a table of the scattering form factors for all ion species that are present at each given timestep. For realistic simulations, the contribution of the inelastic (incoherent Compton) X-ray scattering should be added to the patterns. It is necessary if the energy resolution of the X-ray imaging detector is too low to extract the effect of Compton shift and broadening from the data.[33] This is usually the case for 2D X-ray detectors). The elastic scattering factors and the inelastic structure factors for the atoms and ions were calculated with the XATOM toolkit.[33] As the chosen photon energy was far from all photoionization edges, we neglected the effect of anomalous diffraction.[34]

The simulation details of our study case are as follows. Nitrogenase iron protein (PDB:2NIP, chemical composition: $C_{2717}H_{5151}N_{738}O_{1230}S_{46}Fe_{4}$, weight: 64 kDa) was chosen. As the spatial X-ray intensity gradient appears at a larger length-scale than the size of the sample, spatially homogeneous intensity was assumed within the irradiated molecule. Further, we assumed in all runs that the sample experienced the highest fluence of the pulse (which refers to an experimental scheme in which only scattering patterns containing the largest total photon count are kept for processing). For both 9 fs and 30 fs pulse duration cases, one thousand (1000) radiation damage trajectories were calculated, saving one hundred and fifteen (115) and one

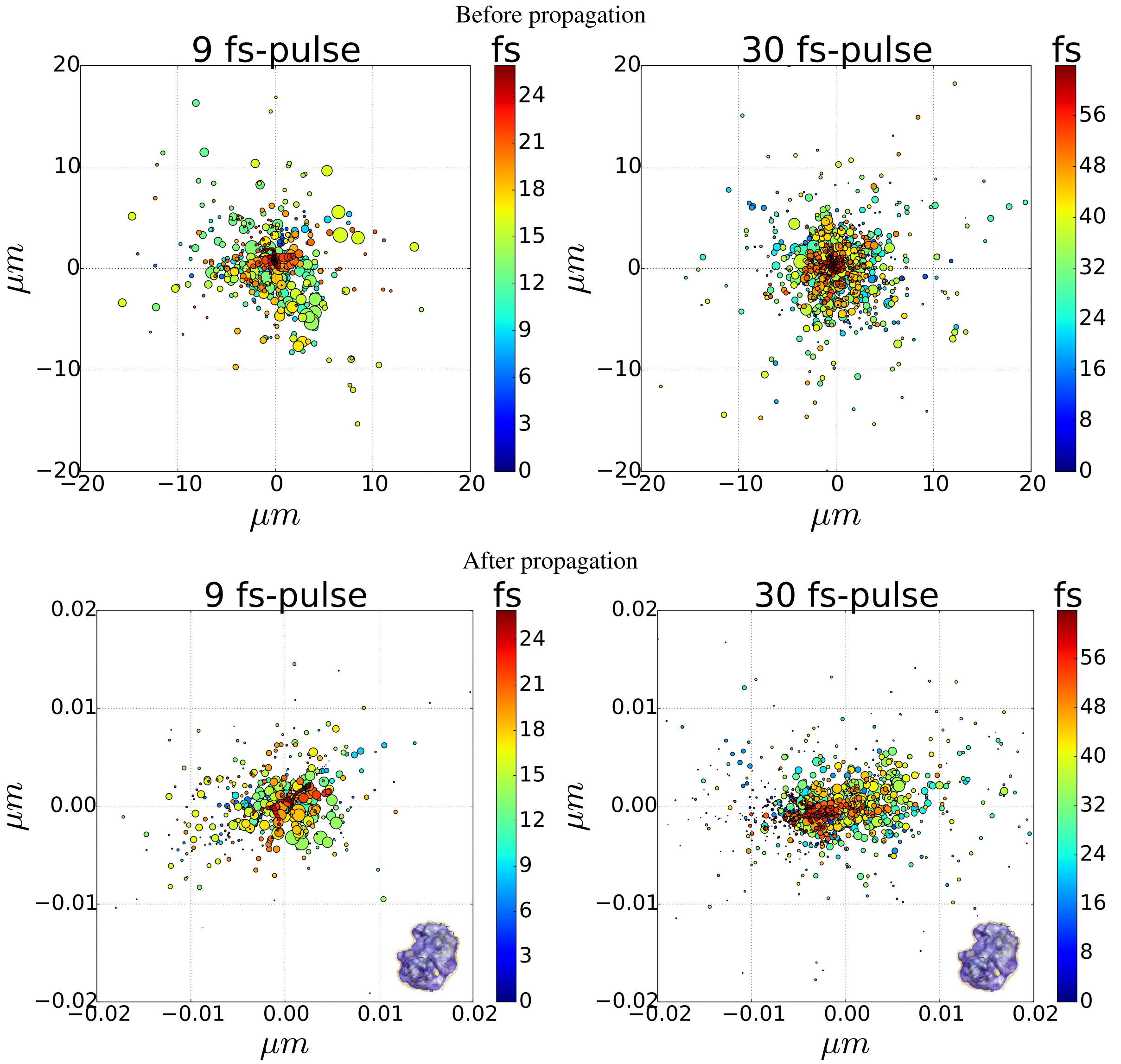

Figure 5. Slice-to-slice shift of the pulse center of mass after propagation through the optics. The slice times are color-coded and the intensities are represented by the size of the circles. A protein molecule of 80 Å is also shown for scale. Note the shift is much smaller than the spot size.

hundred and one (101) intermediate steps respectively. As the number of trajectories is larger than the number of pulse instances, we reused simulated pulses from the PROP module with different random seeds of the Monte Carlo block in XMDYN. The trajectories were propagated for the duration covered by the PROP module output, that was 26 fs and 64 fs respectively. The simulation of one trajectory with 0.78 as timestep took then 1 and 4 hours, respectively, using GPU acceleration on a TESLA 2090 card.

Diffraction

The Diffr module, which calculates the diffraction from a sample and propagates it to a 2D detector, was implemented using the SingFEL software suite.[35]

The weighted mean of the SASE photon energy spectrum of the pulse can be used to shorten the calculation time of the coherent diffraction pattern (rather than using the entire spectrum of the SASE pulse). The weighted mean photon energy $\bar{E}$ is

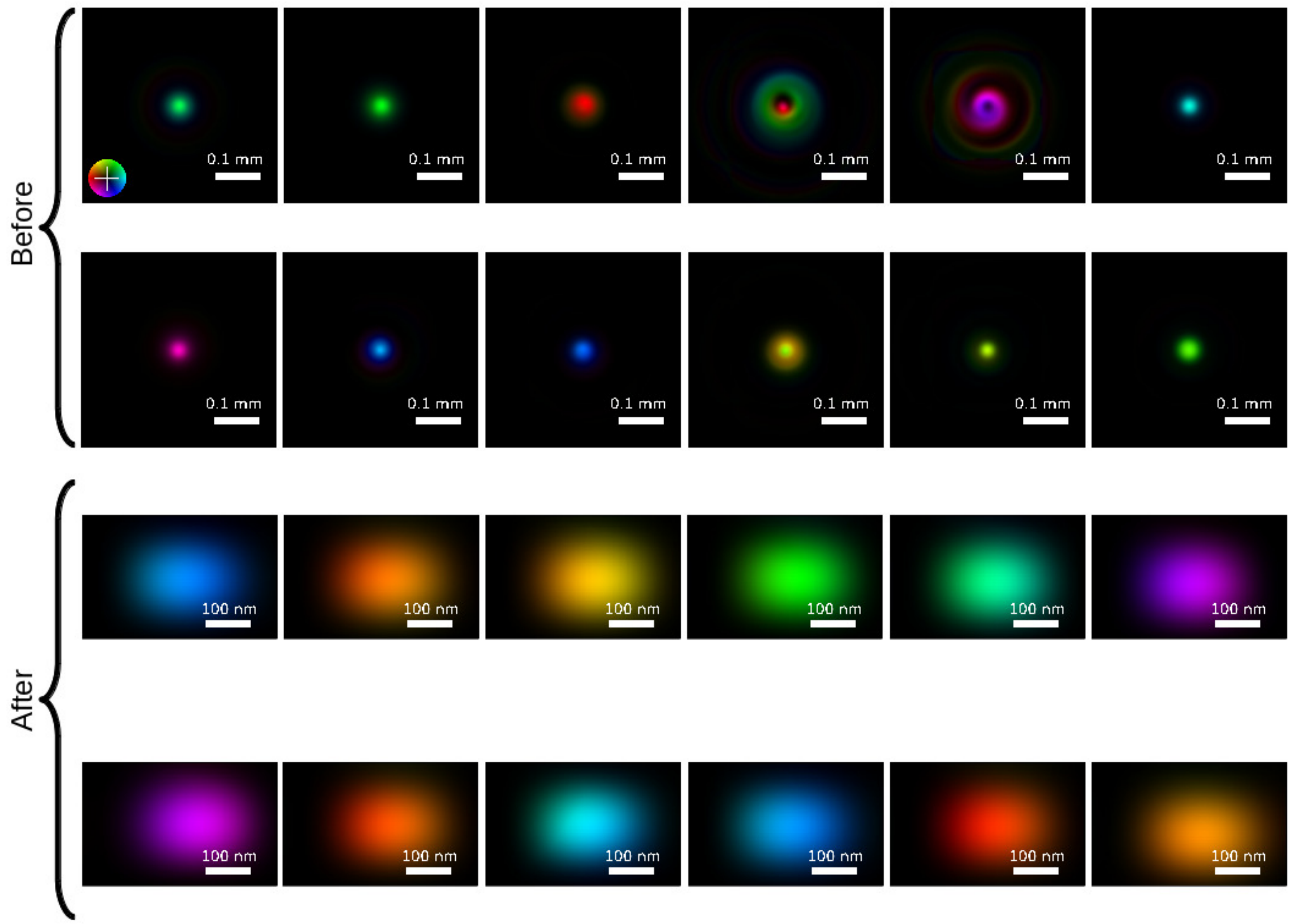

Figure 6. Intensity and phase maps of the SASE FEL X ray slices in a 9 fs pulse before and after propagating through the optics. The phase is color-coded. The distances between slices are about 0.2 fs.

given by

$$\bar{E} = \frac{\sum_{j=-\infty}^{\infty} w_j E_j}{\sum_{j=-\infty}^{\infty} w_j}, \tag{4}$$

where w_j and E_j are the jth weight and photon energy components of the SASE spectrum, respectively. The weighted mean SASE wavelength is then

$$\lambda = \frac{hc}{\bar{E}}. \tag{5}$$

The polarization factor[36] is

$$\cos^2\mu + \sin^2\mu\cos^2 2\theta, \tag{6}$$

where μ is the polarization angle with respect to the horizontal plane and 2θ is the angle between the beam and the observation directions. For horizontally polarized x-ray ($\mu = 0$), the polarization factor is unity. The intensity scattered into a unit solid angle at angle 2θ is

$$\begin{aligned} I_{eTh} &= \frac{r_e^2}{r^2}\left(\cos^2\mu + \sin^2\mu\cos^2 2\theta\right) \\ &= \frac{r_e^2}{r^2}, \end{aligned} \tag{7}$$

where r is the distance between the interaction region to the pixel center and r_e is the classical radius of the electron ($2.818e^{-15}m$)

$$r_e = (\mu_0/4\pi)(e^2/m), \tag{8}$$

where $\mu_0 = 1/(c^2\varepsilon_0)$, e is the electric charge, m is the mass of the electron, c is speed of light and ε_0 is the vacuum permeability.

Hence the intensity scattered into a pixel can be expressed as a differential Thomson scattering cross section per electron

$$\begin{aligned} I_{solid} &= \frac{A_{pix}}{r^2} r_e^2 \left(\cos^2\mu + \sin^2\mu \cos^2 2\theta\right) \\ &= \Omega r_e^2 \left(\cos^2\mu + \sin^2\mu \cos^2 2\theta\right) \\ &= \Omega \frac{d\sigma_T(\theta)}{d\Omega}. \end{aligned} \tag{9}$$

where an analytical formula for the solid angle Ω of a square pixel[37] is

$$\Omega = 4\arcsin\left(\sin^2\alpha\right), \tag{10}$$

and

$$\alpha = \arctan\left(\frac{w}{2r}\right), \tag{11}$$

with w is the pixel width.

The atomic form factors, which in general change as the XFEL propagates through the sample, are input from the PMI module as a function of time t and reciprocal space $\sin\theta/\lambda$. This is consistent with the convention used in Cromer-Mann and Waasmaier-Kirfel coefficients[38]

$$f^0(\sin\theta/\lambda, t) = \sum_{i=1}^{5} a_i(t) \exp\left[-b_i(t)(\sin\theta/\lambda)^2\right] + c, \tag{12}$$

for $0 < \sin\theta/\lambda < 6.0\text{Å}^{-1}$.

From Bragg's Law, $q/2 = \sin\theta/\lambda$ where $q = \sqrt{h^2+k^2+l^2}$ is the reciprocal space modulus. Each detector pixel intersects the 3D reciprocal space (h,k,l) defined by the diffraction geometry. The form factor $f^0(\sin\theta/\lambda)$ is equivalent to $f^0(q/2)$. The structure factor for a given electronic configuration c is

$$F_c(q/2,t) = \sum_{j=1}^{n} f_{cj}^0 \exp\left[2\pi i(hx_{cj} + ky_{cj} + lz_{cj})\right], \tag{13}$$

where f_{cj}^0 is the atomic form factor for the jth atom. Total coherent (Rayleigh) scattering cross section per atom over time is given by

$$\sigma_{coh} = \int_{-\infty}^{\infty} d\sigma_T(\theta) \left|F_c(q/2,t)\right|^2 dt, \tag{14}$$

and total incoherent (bound- and free-electron Compton) scattering cross section per atom over time is defined as

$$\sigma_{inc} = \int_{-\infty}^{\infty} d\sigma_{KN}(\theta) \left[S_c(q/2,t) + N_c^{free}(t)\right] dt, \tag{15}$$

where S_c is the Compton scattering factor and N_c^{free} is the inelastic scattering from free electrons. For X-rays, the differential Klein-Nishina (free-electron Compton) collision cross section per electron [39,40] can be simplified to

$$\frac{d\sigma_{KN}(\theta)}{d\Omega} = \frac{d\sigma_T(\theta)}{d\Omega}. \tag{16}$$

Therefore, for a polarized X-ray pulse interacting with a molecule in some initial electronic configuration c over time, the mean number of scattered photons I_c arriving at a detector pixel at position $q/2$ of projected solid angle Ω from both coherent and incoherent scattering is given by eqn. 17.

$$I_c(q/2,\Omega) = \Omega \frac{d\sigma_T(\theta)}{d\Omega} \int_{-\infty}^{\infty} I_i(t) \left(\left|F_c(q/2,t)\right|^2 + S_c(q/2,t) + N_c^{free}(t)\right) dt, \tag{17}$$

where $I_i(t)$ is the number of incident photons per unit area. The diffracted intensity is sampled according to Poisson statistics. A circular beamstop and a horizontal detector gap masks the corresponding pixels. For coherent scattering only simulation, $S_c(q/2,t)+N_c^{free}(t)$ is removed in eqn. 17. Note that the photon energy from the incoherent scattering would deviate slightly from the incoming photon energy, which is not accounted for in this module. This effect is, however, negligible, as such a small energy difference is in practice not detectable by 2D area detectors used for CDI.

In our case study, a single diffraction pattern of an exploding protein, as seen by the detector, is calculated as the incoherent sum of diffraction patterns at each instance in time during the entire duration of the XFEL pulse. In order to keep the calculation tractable, we (1) use the mean photon energy of the X-ray pulse instead of the entire SASE spectrum, (2) downsample the effective detector pixel size, (3) generate multiple diffraction patterns per PMI trajectory, and (4) decrease the number of temporal samples of the radiation damage process.

The mean photon energy is defined in eq. 4, and this simplification is expected to have little effect on the validity of the simulations for photon energies away from absorption edges of atoms in the sample. Near edges, the full spectrum could be used at the expense of computational time.

We derive the speckle width in the reconstructed intensity to determine the feasibility of downsampling the effective detector pixel size. The maximum scattering angle to the edges of the detector θ_{max} and the scattering angle subtended by a single pixel θ_{pix} are given by

$$\theta_{max} = \arctan\left(\frac{pn/2}{d}\right), \tag{18}$$

and

$$\theta_{pix} = \arctan\left(\frac{p}{d}\right), \tag{19}$$

where n is the number of pixels along the edge of the detector, p is the pixel size and d is the sample to detector distance. In physics, the maximum scattering vector q_{max} and the half period resolution d_{min} are defined as

$$q_{max} = \frac{4\pi}{\lambda}\sin\left(\theta_{max}/2\right), \tag{20}$$

and

$$d_{min} = \frac{2\pi}{2q_{max}}. \tag{21}$$

It is conventional to omit the factor of 2π in crystallographic definitions of q_{max} and d_{min}. The speckle width is defined as

$$\Delta q_{speckle} = \frac{2\pi}{w}. \tag{22}$$

Reciprocal spacing per voxel is given by

$$\Delta q = \frac{q_{max}}{p_{max}}, \tag{23}$$

where $p_{max} = floor\left[\theta_{max}/\theta_{pix}\right]$ is the half number of voxels along the edge of the reconstructed intensity. Finally, the number of pixels per speckle width is given by

$$n_{speckle} = \frac{\Delta q_{speckle}}{\Delta q}. \tag{24}$$

In the simulations, we set the detector distance d to 13 cm (which is the minimum distance at the SPB/SFX endstation). The AGIPD detector's pixel size p is 200 μm. We downsample by a factor of 6 so the effective pixel size is 1200 μm. The simulated detector has 81×81 effective pixels which sets the detector resolution at the edge to 3.5Å at 5 keV. This sampling change reduces the number of pixels per speckle width (eq. 24) to 3.4 pixels which is still greater than the required oversampling of 2.

We sample every 10th time slice of a 9 fs FDHM pulse (1.99 fs interval) and every 3rd time slice of a 30 fs FDHM pulse (2.29 fs interval). From 1,000 PMI trajectories, we calculate 200 independent diffraction patterns by randomly rotating the sample (i.e. a total of 200,000 patterns each) in SO(3). We use eq. 17 to calculate the diffraction patterns. The sparseness of the photons arriving at the detector is shown in Supplementary Fig. S7. Simulations for 9 fs and 30 fs cases took 4 days and 9 days, respectively with 63 parallel jobs. If the radiation-induced atomic movements were not significant, an effective scattering factor introduced in[41] could have been used to shorten the computation time.

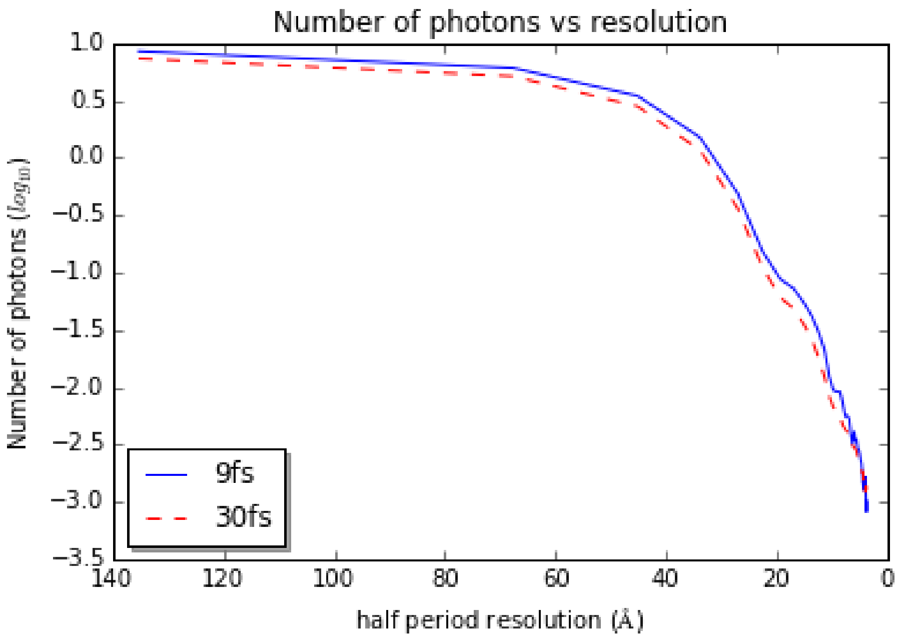

Figure 7. Number of photons vs resolution for 9 fs and 30 fs pulse cases.

Orientation recovery

EMC[42] is an iterative, *de novo* reconstruction of a three-dimensional (3D) diffraction volume $\mathbf{W} = \{W_j\}$, comprising Ewald-sphere sections sampled at orientations j, that is most compatible with a set of 2D diffraction patterns $\mathbf{K} = \{K_k\}$. It determines the compatibility between each K_k and each W_j via a Poissonian log-likelihood function $Q(K_k|W_j)$. In the EMC algorithm, this is done in three iterative steps. An Expansion step projects the diffraction volume into Ewald sphere sections ($\mathbf{W} \to W_j|\mathbf{K}$) quasi-uniformly distributed over the SO(3) rotation group. The Maximization step that follows maximizes the expectation of individual Ewald sections given the collected patterns $\mathbf{K}$ via this likelihood function ($W_j \to \widetilde{W}_j$). Finally, the Compression step inserts the Ewald sections back into an updated 3D diffraction volume ($\widetilde{W}_j \to \widetilde{\mathbf{W}}$).

Our implementation of EMC uses the Poissonian log-likelihood function in,[42] which were also discussed plainly with two minimal models.[43,44] This is appropriate because our diffraction patterns are photon-limited with no explicit spurious background noise. More sophisticated implementations of EMC can recover the pulse-to-pulse intensities,[45] particle conformations[46] and deal with spurious background photons.[44]

We reconstruct the 3D diffraction volume with progressively finer rotation group sampling, up to 36540 quasi-uniformly distributed quaternions in SO(3).[42] The progress of the reconstructions is monitored through the numerical change in the updated diffraction volume in Supplementary Fig. S8. Each time this numerical change falls below a threshold of 4×10^{-8} per voxel, the reconstruction would proceed with a finer rotation group sampling ('quat' labels in Supplementary Fig. S8 mark these refinements). A single reconstruction attempt terminates when this threshold is breached at the finest rotation group sampling or a maximum number of iterations has been performed, whichever occurs sooner. Typical single reconstructions in this paper took around 30 hours to finish on eight Intel Xeon X7542 2.6 GHz cores running an OpenMP implementation of EMC.

Planar sections of the iteratively reconstructed 3D diffraction volume are informative diagnostics (Supplementary Fig. S9). From these we can detect if reconstructions are resolution-limited, when intensity speckles have been defined, and if its speckle contrast is sufficient for phase-retrieval. Sections of earlier iterations in Supplementary Fig. S9 tend to be more rotationally averaged. The effects of the missing data regions in the detector (Fig. 8) on the assembled diffraction volume is also apparent in low spatial frequencies of Supplementary Fig. S9.

The top panel of Supplementary Fig. S10 shows another diagnostic of EMC, which measures how often each diffraction pattern's most likely orientation changes within the diffraction volume with each iteration. An important caveat: in EMC each diffraction pattern K is added to new diffraction volume $\widetilde{W}$ not only at the most likely orientation j', but across all orientations j appropriately weighted by the conditional probability that it arises from each orientation in the current model W. This probablistic interpretation of orientations is typical of Bayesian algorithms. Hence, Supplementary Fig. S10 is merely a simplified proxy to visualize the extent that each pattern's conditional probability has been resolved by looking its most likely

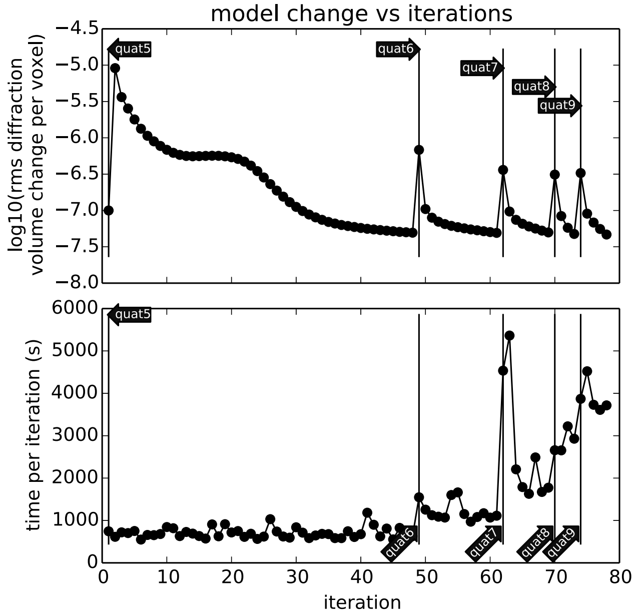

Figure 8. Numerical convergence of the *de novo* reconstruction of a scatterer's three-dimensional diffraction volume, as the rotation group sampling of this volume is gradually increased (e.g. quaternion refinement level $5 \to 9$). The fluctuations in the iteration time are due to an optimization in EMC's Maximize step: each pattern is only added to the new diffraction volume at orientations that are significant in the current diffraction volume.

orientation.

Finally, the bottom panel of Supplementary Fig. S10 shows how the average mutual information per pattern changes with iteration. This plot, though more technical, has a simple diagnostic interpretation. The stability of this mutual information plot implies that the conditional probabilities for the datasets **K** have converged. Notice that even though the error metric in Supplementary Fig. S8 has converged near iteration 40, the fluctuations on the mutual information in Supplementary Fig. S10 informs us that the likelihood of model W is still struggling to accommodate the datasets within a limited number of orientations. Only when the rotation group sampling increases do these fluctuations damp out in Supplementary Fig. S10.

Phase retrieval

In the Born approximation, the coherent scattering measured on our detector is derived from the scatterer as if it imparts only a pure phase on the incident x-ray plane wave, shown in Eq. (14). Combining this with Eq. (13), the assembled 3D diffraction intensities I_{coh} are equivalent to the Fourier intensities of the scatterer's electron density function. Phase retrieval algorithms iteratively update a guess of the electron density $x(\mathbf{r})$ by composing two projection operators in a specific recipe. First, the Fourier projection operator projects $x(\mathbf{r})$ to satisfy the constraint $|\widehat{F}[x(\mathbf{r})]| \to \sqrt{I_{\text{coh}}}$ via

$$\widehat{P}_F\left[x(\mathbf{r})|\sqrt{I_{\text{coh}}}\right] = \widehat{F}^{-1}\left[\begin{cases}\sqrt{I_{\text{coh}}}\frac{\widehat{F}[x(\mathbf{r})]}{|\widehat{F}[x(\mathbf{r})]|}, & \text{if } I_{\text{coh}} > 0\\ \widehat{F}[x(\mathbf{r})], & \text{otherwise}\end{cases}\right], \tag{25}$$

where $\widehat{F}[\cdot]$ and $\widehat{F}^{-1}[\cdot]$ are the Fourier transform and inverse Fourier transform operators. The second projection operates in real space, by projecting $x(\mathbf{r})$ to real-valued densities within a compact support S:

$$\widehat{P}_S\left[x(\mathbf{r})|S\right] = \begin{cases}x(\mathbf{r}), & \text{if } \mathbf{r} \in S \text{ and } x(\mathbf{r}) \in \mathbf{R}\\ 0, & \text{otherwise}\end{cases}. \tag{26}$$

Phase retrieval seeks the family of solution electron distribution function $\widetilde{x}(\mathbf{r})$ that satisfy both the Fourier and real space constraints, or more formally, the case when $\delta \to 0$ in

$$\left|\widehat{P}_F\left[\widehat{P}_S[\widetilde{x}(\mathbf{r})]\right] - \widetilde{x}(\mathbf{r})\right| = \delta. \tag{27}$$

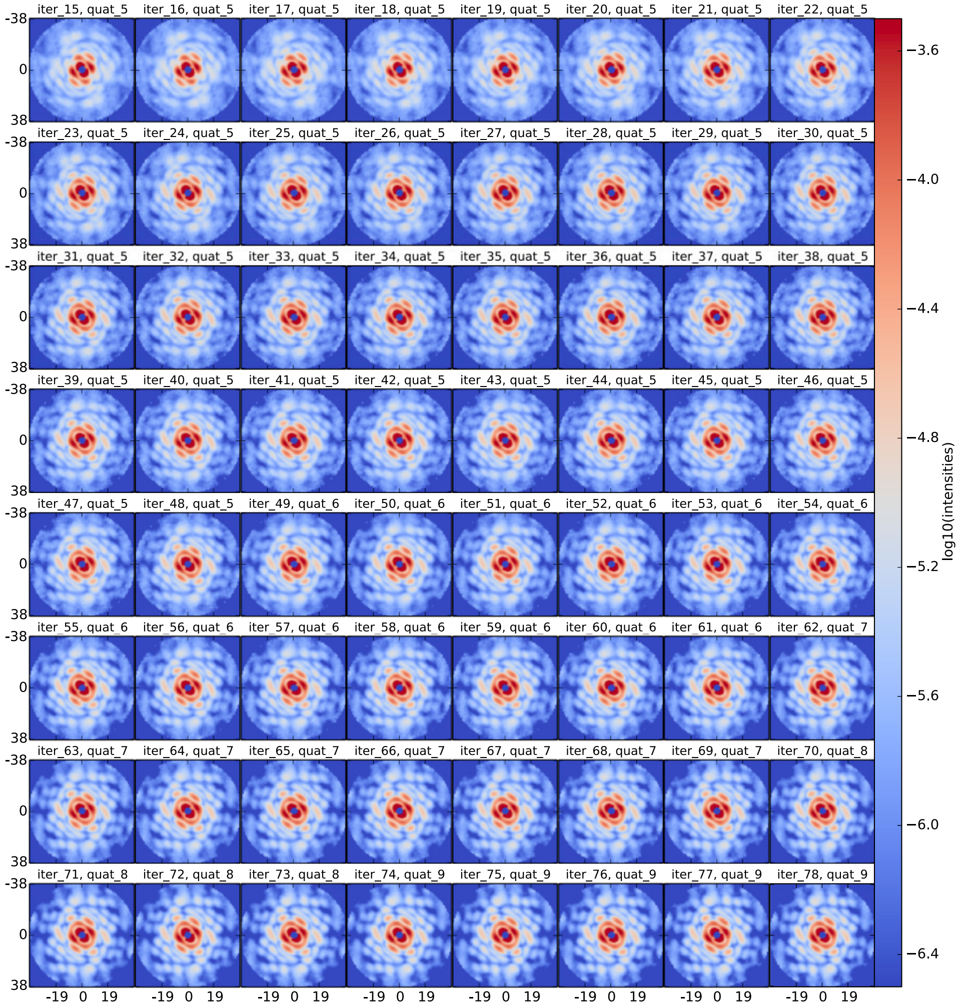

Figure 9. Central slices of iteratively reconstructed three-dimensional diffraction volume. Each section is labeled by the dimensionless spatial frequencies, iteration numbers and rotation group sampling (quat5-9).

In case where the Fourier and real space constraints become incompatible such that $\delta > 0 \forall x(\mathbf{r})$ in Eq. (27), the search for $\widetilde{x}(\mathbf{r})$ may not succeed altogether. This occurs when incoherent scattering from free and bound electrons are added to the diffraction patterns via Eq. (17), or with photon shot-noise, or when the 2D diffraction from differently damaged scatterers are incoherently added together to produce assembled 3D intensities. These cause a mismatch in the Fourier reciprocity between measured diffraction I_{meas} and desired electron densities, quantified by δ in Eq. (27). Furthermore, typically the support S in $\widehat{P}_S$ is initially unknown and estimated with the autocorrelation support obtained from the diffraction intensities $\widehat{F}[I_{\text{meas}}]$.[47] Overall, the mismatch between I_{meas} and I_{coh} can destabilize phase retrieval algorithms.

For sufficiently small mismatches, a lower-resolution estimate of undamaged density can still be recovered. One method for doing so is the modified Elser's Difference Map,[48] which iteratively advances towards the solution $\widetilde{x}(\mathbf{r})$ via the update

$$x_{n+1}(\mathbf{r}) = x_n(\mathbf{r})' + \varepsilon_n \tag{28}$$

where

$$x_n(\mathbf{r})' = \alpha x_n(\mathbf{r}) + (1-\alpha)\widehat{P}_F[x_n(\mathbf{r})] \tag{29}$$

$$\varepsilon_n = \widehat{P}_F\left[2\widehat{P}_S[x_n(\mathbf{r})'] - x_n(\mathbf{r})'\right] - \widehat{P}_S[x_n(\mathbf{r})'] \quad . \tag{30}$$

In our implementation we have optimized $\alpha = 0.2$. Notably, $\alpha = 0$ reduces to a special case of the Difference Map.[49]

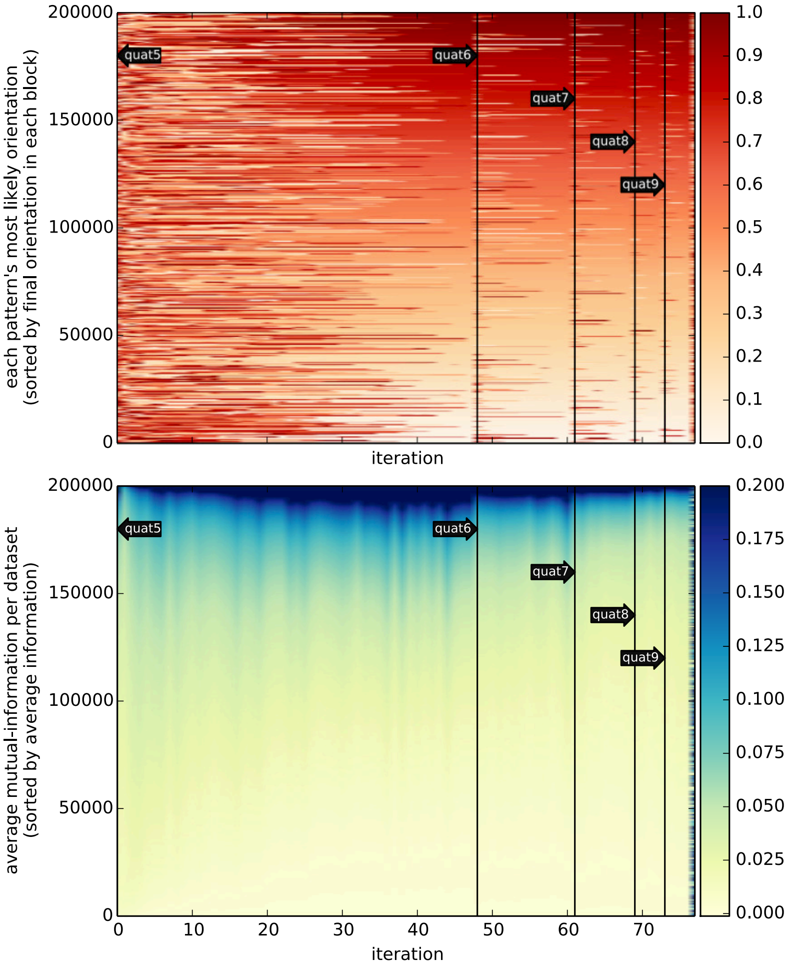

Figure 10. Orientation certainty and mutual information of individual patterns given an iteratively improved 3D diffraction volume. **Top:** diffraction patterns are sorted within each rotation sampling block (quat*) by the orientation index of the final iteration within that block. Orientations are indicated on a gradient color scale. As the reconstructed diffraction volume converges, so do the most likely orientations of each pattern. **Bottom:** Average mutual information of each pattern across all orientations, divided by the entropy of the rotation group sampling. While a value of unity indicates perfect orientation certainty, this is also typical of overfitting to noisy and incomplete data. Patterns with zero mutual information have completely unknown orientations. Unlike the top panel, values on the vertical axes do not label specific patterns because we separately sorted the columns by the mutual information per pattern for each iteration.

Ideally, the search in Eq. (28) should terminate only when ε_n in Eq. (30) vanishes, but because of the constraint mismatch above we consider the search to have converged when ε_n takes on the smallest value within a fixed number of iterations.

Realistically, our goal in iterative phase retrieval is to find reproducible features that are constrained by the assembled measurements. It is non-trivial to determine if a set of noisy assembled 3D intensities has a unique solution electron density. We adopted the following approach that avoids over-fitting, and also averages away transient, spurious features.

1. Obtain the autocorrelation support A by applying two-means clustering of $\widehat{F}[I_{\text{meas}}]$. We uniformly contract A by a factor of two to obtain the initial support S_0.

2. Repeat this sub-routine one hundred times :

   - Let S_i be the most updated support. Initialize $x_0(\mathbf{r})$ on a cubic array with random numbers.
   - Run iterative phase retrieval described in Eq. (28) for 500 iterations, and save the Fourier estimate

$$E' = \widehat{P}_F\left[2\widehat{P}_S[x_n(\mathbf{r})'] - x_n(\mathbf{r})'\right]$$

with the smallest ε_n in Eq. (30). In a similar bit-retrieval problem studied in,[50] this selection criteria can yield phases closest to the correct one even when the measurements are very noisy.

3. These five hundred $\{E'\}$ from randomly initialized $x_0(\mathbf{r})$ in step 2 are averaged to give the electron distribution functions $\langle E'(\mathbf{r})\rangle_{500}$.

4. We threshold $\langle E'(\mathbf{r})\rangle_{500}$ at the 7% level and convolve it with a $5\times5\times5$-voxel volume to update the support from $S_i \to S_{i+1}$.

5. Repeat steps 2-4 until $\langle E'(\mathbf{r})\rangle_{500}$ changes minimally between repetitions. The reconstructions in Fig. 7 in the main text were the resultant $\langle E'(\mathbf{r})\rangle_{500}$ at the end of ten such repeats.

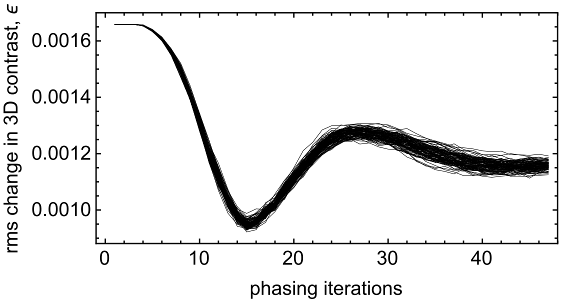

Figure 11. The plot of ε in Eq. (30) in a hundred randomly initialized phasing reconstructions (super-imposed lines). Fourier estimates from the iterate with the lowest ε in each reconstruction trial are added together to produce the average densities in Fig. 7 in the main text.

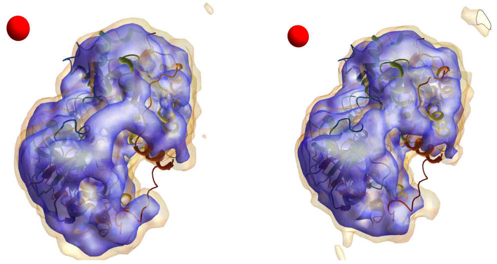

Figure 12. Reconstructions in the 9 fs case (with Compton scattering), processed with two different shrinkwrap trajectories. Starting from S_0 derived from the autocorrelation support, the support was independently refined from two ensembles of randomly initialized reconstructions using the prescription described above. Electron densities of the average reconstruction at the 5% and 15% levels (orange and blue, respectively) are rendered in both cases.

References

1. Saldin, E. L., Schneidmiller, E. A. & Yurkov, M. V. *The Physics of Free Electron Lasers* (Springer-Verlag, Berlin, 1999).

2. Saldin, E. L., Schneidmiller, E. A. & Yurkov, M. V. The general solution of the eigenvalue problem for a high-gain fel. *Nucl. Instrum. and Methods* **A**, 86 (2001).

3. Schneidmiller, E. A. & Yurkov, M. V. Harmonic lasing in x-ray free electron lasers. *Phys. Rev. ST Accel. Beams* **15**, 080702 (2012).

4. Saldin, E. L., Schneidmiller, E. A. & Yurkov, M. V. Diffraction effcts in the self-amplifed spontaneous emission FEL. *Opt. Commun.* **186**, 185 (2000).

5. Schneidmiller, E. A. & Yurkov, M. V. Coherence properties of the odd harmonics of the radiation from sase fel with planar undulator. http://accelconf.web.cern.ch/AccelConf/FEL2012/papers/mopd08.pdf (2012) (Date of access:09/02/2016).

6. Penman, C. & McNeil, B. W. J. Simulation of input electron noise in the free-electron laser. *Optics Comm.* **90**, 82–84 (1992).

7. Fawley, W. M. Algorithm for loading shot noise microbunching in multidimensional, free electron laser simulation codes. *Phys. Rev. STAB* **5**, 070701 (2002).

8. Saldin, E. L., Schneidmiller, E. A. & Yurkov, M. V. Coherence properties of the radiation from X-ray free electron laser. *Opt. Commun.* **281**, 1179 (2008).

9. Altarelli, M. *et al.* XFEL: The European X-Ray Free-Electron Laser. Technical Design Report. *Preprint DESY 2006-097* (2006).

10. Tschentscher, T. Layout of the X-Ray Systems at the European XFEL. *Technical Report* DOI:10.3204/XFEL.EU/TR–2011–001 (2011).

11. Decking, W. & Limberg, T. European XFEL Post-TDR Description. *Technical Note* http://xfel.eu/documents/technical_documents (2013) (Date of access:09/02/2016).

12. Pflüger, J. *et al.* Status of the Undulator Systems for the European X-ray Free Electron Laser. *Proc. FEL2013, TUPS060* 367–371 (2013).

13. Manetti, M. *et al.* XFEL Photon Pulses Database (XPD) for modeling XFEL experiments. http://in.xfel.eu/xpd (2015) (Date of access:09/02/2016).

14. Schneidmiller, E. & Yurkov, M. An overview of the radiation properties of the European XFEL. *Proc. FEL2014, MOP066* 204–209 (2014).

15. Schropp, A. & Schroer, C. G. Dose requirements for resolving a given feature in an object by coherent x-ray diffraction imaging. *New Journal of Physics* **12**, 035016 (2010).

16. Slowik, J. M., Son, S.-K., Dixit, G., Jurek, Z. & Santra, R. Incoherent x-ray scattering in single molecule imaging. *New Journal of Physics* **16**, 073042 (2014).

17. Williams, G. J., Quiney, H. M., Peele, A. G. & Nugent, K. A. Coherent diffractive imaging and partial coherence. *Phys. Rev. B* **75**, 104102 (2007).

18. Schneidmiller, E. & Yurkov, M. Photon Beam Properties at the European XFEL. *Preprint DESY 11-152, DESY, Hamburg* DOI:10.3204/DESY11-152 (2011).

19. Chubar, O. *et al.* Time-dependent FEL wavefront propagation calculations: Fourier optics approach. *Nuclear Instruments and Methods in Physics Research A* **593**, 30–34 (2008). URL http://www.sciencedirect.com/science/article/pii/S0168900208006177.

20. Samoylova, L., Buzmakov, A., Chubar, O. & Sinn, H. WavePropaGator: Interactive framework for X-ray FEL optics design and simulations. Submitted to J.Appl.Cryst.

21. Rutishauser, S. *et al.* Exploring the wavefront of hard x-ray free-electron laser radiation. *Nat Commun* **3**, 947 (2012). URL http://dx.doi.org/10.1038/ncomms1950.

22. Canestrari, N., Chubar, O. & Reininger, R. Partially coherent x-ray wavefront propagation simulations including grazing-incidence focusing optics. *J. Syn. Rad* **21**, 1110–1121 (2014).

23. Murphy, B. F. *et al.* Femtosecond X-ray-induced explosion of C60 at extreme intensity. *Nat. Commun.* **5**, 4281 (2014).

24. Berrah, N. *et al.* Emerging photon technologies for probing ultrafast molecular dynamics. *Faraday Discuss* **171**, 471–485; DOI:10.1039/c4fd00015c (2014).

25. Tachibana, T. *et al.* Nanoplasma Formation by High Intensity Hard X-rays. *Scientific Reports* **5**, 10977 (2015).

26. Jurek, Z., Ziaja, B. & Santra, R. Applicability of the classical molecular dynamics method to study x-ray irradiated molecular systems. *J. Phys. B* **47**, 124036 (2014).

27. O'Boyle, N. M. *et al.* Open Babel: An open chemical toolbox. *J. Cheminf.* **3**, 33; DOI:10.1103/PhysRev.81.385 (2011).

28. The open babel package (2014). Version 2.3.2 http://openbabel.org.

29. Son, S.-K., Young, L. & Santra, R. Impact of hollow-atom formation on coherent x-ray scattering at high intensity. *Phys. Rev. A* **83**, 033402; DOI:10.1103/PhysRevA.83.033402 (2011).

30. Slater, J. C. A Simplification of the Hartree-Fock Method. *Phys. Rev.* **81**, 385–390; DOI:10.1103/PhysRev.81.385 (1951).

31. Qian, X. & Schlick, T. Efficient multiple-time-step integrators with distance-based force splitting for particle-mesh-ewald molecular dynamics simulations. *J. Chem. Phys* **116**, 5971–83 (2002).

32. Kim, Y. & Rudd, M. Binary-encounter-dipole model for electron-impact ionization. *Phys. Rev. A* **50**, 3954 (1994).

33. Slowik, J. M., Son, S.-K., Dixit, G., Jurek, Z. & Santra, R. Incoherent x-ray scattering in single molecule imaging. *New J Phys.* **16**, 073042 (2014).

34. Son, S.-K., Chapman, H. N. & Santra, R. Multiwavelength Anomalous Diffraction at High X-Ray Intensity. *Phys. Rev. Lett.* **107**, 218102 (2011).

35. Yoon, C. H. *et al.* SingFEL: single particle imaging package. http://singfel.readthedocs.org (2015) (Date of access:09/02/2016).

36. Giacovazzo, C. *et al. Fundamentals of Crystallography* (IUCr/Oxford University Press, 2014), third edn.

37. Zaluzec, N. J. Analytical formulae for calculation of x-ray detector solid angles in the scanning and scanning/transmission analytical electron microscope. *Microsc. Microanal.* **20**, 1318–1326 (2014).

38. Waasmaier, D. & Kirfel, A. New Analytical Scattering Factor Functions for Free Atoms and Ions for Free Atoms and Ions. *Acta Cryst.* **A**, 416–413 (2014).

39. Slowik, J. D., Son, S.-K., Dixit, G., Jurek, Z. & Santra, R. Incoherent x-ray scattering in single molecule imaging. *New Journal of Physics* **16**, 073042 (2014).

40. Jurek, Z., Thiele, R., Ziaja, B. & Santra, R. Effect of two-particle correlations on x-ray coherent diffractive imaging studies performed with continuum models. *Phys. Rev. E* **86**, 036411 (2012).

41. Lunin, V. Y. *et al.* Efficient calculation of diffracted intensities in the case of nonstationary scattering by biological macromolecules under XFEL pulses. *Acta Cryst.* **D**, 293–303 (2015).

42. Loh, N.-T. D. & Elser, V. Reconstruction algorithm for single-particle diffraction imaging experiments. *Phys. Rev. E* **80**, 6705 (2009).

43. Elser, V. Noise Limits on Reconstructing Diffraction Signals From Random Tomographs. *Inf. Theory, IEEE Trans.* **55**, 4715–4722 (2009).

44. Loh, N. D. A minimal view of single-particle imaging with X-ray lasers. *Philos. Trans. R. Soc. B Biol. Sci.* **369**, 20130328 (2014).

45. Loh, N. D. *et al.* Cryptotomography: Reconstructing 3D Fourier Intensities from Randomly Oriented Single-Shot Diffraction Patterns. *Phys. Rev. Lett.* **104**, 25501 (2010).

46. Loh, N. D. Effects of extraneous noise in Cryptotomography. *Proc. SPIE* **8500** (2012).

47. Marchesini, S. *et al.* X-ray image reconstruction from a diffraction pattern alone. *Phys. Rev. B* **68**, 140101 (2003).

48. Loh, N.-T. D., Eisebitt, S., Flewett, S. & Elser, V. Recovering magnetization distributions from their noisy diffraction data. *Phys. Rev. E* **82**, 61128 (2010).

49. Elser, V. Phase retrieval by iterated projections. *J. Opt. Soc. Am. A* **20**, 40–55 (2003).

50. Elser, V. & Eisebitt, S. Uniqueness transition in noisy phase retrieval. *New J. Phys.* **13**, 023001 (2011).
